# Establishment and Optimization of a Human Flow-Based Hollow Fiber *In Vitro* Blood–Brain Barrier Model for Systemic Inflammatory Responses

**DOI:** 10.3390/pharmaceutics18070817

**Published:** 2026-06-30

**Authors:** Anna Gerhartl, Maria Kirchsteiger, Andreas Brachner, Lena Czeloth, Barbora Valentova, Iola F. Duarte, Winfried Neuhaus

**Affiliations:** 1 Competence Unit Molecular Diagnostics, Center Health and Bioresources, AIT–Austrian Institute of Technology GmbH, Giefinggasse 4, 1210 Vienna, Austriaandreas.brachner@ait.ac.at (A.B.);; 2 Department of Chemistry, LAQV-REQUIMTE, University of Aveiro, 3810-193 Aveiro, Portugal; ioladuarte@ua.pt; 3 Department of Medicine, Faculty of Medicine and Dentistry, Danube Private University, 3500 Krems, Austria

**Keywords:** DIV model, BBB, hCMEC/D3, astrocytes, pericytes, shear stress, cytokine, long term, NMR spectroscopy, metabolomics

## Abstract

**Background/Objectives**: Systemic inflammation and circulating proinflammatory cytokines can impair blood–brain barrier (BBB) integrity. Many *in vitro* BBB models lack the complexity to fully recapitulate systemic inflammation and its long-term effects on the BBB. This study aimed to develop a hollow-fiber flow-based dynamic *in vitro* (DIV) BBB model for investigating prolonged proinflammatory responses under physiological flow conditions. **Methods**: Culture conditions for hCMEC/D3 in a DIV model (Flocel) were optimized by varying serum concentrations over seven weeks. Barrier integrity (transendothelial electrical resistance (TEER), permeability studies), proliferation, metabolism (NMR spectroscopy) and molecular changes (high-throughput qPCR) were assessed. Optimized triple-cultures with hCMEC/D3, human primary astrocytes and pericytes were established. After four weeks of barrier establishment, the triple-cultures were exposed to TNF-α, IL-1β and IFN-γ (0.1 ng/mL or 10 ng/mL each) for two weeks. The inflammatory response was assessed with a multiplex cytokine array. **Results**: Reduced serum concentration (0.25% FBS) decreased proliferation, promoted aerobic respiration, and altered tight junction and transporter gene expression, accompanied by moderately improved barrier integrity compared with 1% or 5% FBS. In optimized triple-cultures, cytokine exposure induced concentration-dependent secretion of IL-6, IL-8, and MCP-1 and changes in mRNA levels, with minor effects on barrier integrity. Sustained cytokine release over two weeks demonstrated stable induction of inflammatory responses at the BBB. **Conclusions**: An organotypic DIV model of the human BBB, incorporating hCMEC/D3, human primary astrocytes and pericytes was successfully established. By enabling long-term exposure to physiologically relevant cytokine concentrations under flow conditions, this model may provide a platform to investigate functional and molecular BBB responses in inflammation-driven disease progression.

## 1. Introduction

Peripheral injury, disease or bacterial infection can lead to systemic inflammation, causing functional alterations or breakdown of the blood–brain barrier (BBB) [1]. Systemic inflammation itself enhances the synthesis of circulating proinflammatory cytokines in the bloodstream, such as TNF-α, IL-1β, IFN-γ, and IL-6, which, in turn, can affect BBB integrity by eliciting an inflammatory response in cells, such as brain capillary endothelial cells (BCEC), astrocytes, and pericytes [2,3]. Thus, these cell types can play a central role in inflammation by either propagating the disease by the secretion of proinflammatory cytokines (IL-6, IL-8, MCP-1) or counteracting inflammation by the expression of anti-inflammatory cytokines (IL-4, IL-10), depending on intercellular signals from the bloodstream or brain parenchyma (TNF-α, IL-1β or INF-γ) [1,4,5]. The BBB separates the brain parenchyma from the bloodstream by serving as a physical, metabolic and transport barrier to limit the entry of harmful substances, signaling molecules and pathogens that could impair or alter brain function. The physical barrier of the BBB is established by BCEC, the main cellular component of the BBB, which is interlinked by junction complexes consisting of a distinct set of tight junction (TJ) proteins and adherens junction (AJ) proteins (e.g., VE-cadherin linked to b-catenin intracellularly). The tight junction complexes are reported to consist mainly of CLDN-1, -3, -5, -11 and -12 bound to occludin and zonula occludens 1 (ZO-1), as well as tricellulin and LSR (angulin-1), which regulate the formation, assembly, and structure of the tight junction complexes [6,7,8,9]. BCEC also express a distinct set of transporter proteins that belong to the ABC (ATP-binding cassette) or the SLC (solute carrier) transporter family, including ABCB1 (PgP, P-glycoprotein), ABCG2 (BCRP, breast cancer resistance protein), ABCC1–5 (MRP1–5, multidrug-resistance related proteins), SLC1A1 (EAAT-3) and SLC2A1 (Glut-1). These transporter proteins act as influx or efflux pumps to control the transport of nutrients, essential amino acids, signaling molecules, and harmful substances across the BBB, thereby forming the transport barrier [7]. The metabolic barrier is formed by enzymes such as cytochrome P450, which facilitate the biotransformation of lipids and steroidal hormones in BCEC before these molecules can enter the brain parenchyma [10,11]. To meet the high energy demand needed to fulfill these barrier functions, BCEC not only use glucose but also glutamine and fatty acids as their main carbon sources in energy-producing metabolic reactions. By glycolysis, glucose is converted into pyruvate, which is further processed depending on oxygen availability. In the absence of oxygen, anaerobic respiration converts pyruvate into lactate, producing two ATP molecules per pyruvate. Under aerobic conditions, pyruvate is converted into acetyl-CoA, which enters the TCA cycle in the mitochondria, driving ATP production. Together, glycolysis and the TCA cycle can generate up to 36 ATP molecules per glucose molecule [12,13,14]. Glutamine is converted into glutamate and subsequently into α-ketoglutarate, which enters the TCA cycle to replenish carbon intermediates (anaplerosis) [15,16,17]. Fatty acids are oxidized via β-oxidation to produce acetyl-CoA, which can directly enter the TCA cycle [14]. Disruption in nutrient availability to BBB-associated or adjacent cells, such as astrocytes, pericytes and neurons, can significantly affect the barrier properties of BCEC [7]. Moreover, pericytes and astrocytes also play a critical role in disease progression, as they contribute to pathophysiological conditions, including neuroinflammation, by expressing proinflammatory cytokines such as IL-1β, IL-6, IL-8, MCP-1 and TNF-α. Additionally, active pericytes and astrocytes can also contribute to the upregulation of VEGF in BCEC and can directly increase BBB leakiness by expressing BBB-degrading enzymes, such as metalloproteinases (MMPs). These enzymes affect the tight junction complexes that seal the paracellular gaps between BCEC [18,19,20,21,22,23].

Furthermore, shear stress induced by blood flow along the BCEC influences their morphological, metabolic, and transcriptomic properties. It is estimated that *in vivo* BCEC are exposed to a shear stress of 5–20 dyne/cm^2^ [24,25,26,27,28,29]. To mimic the complexity of the BBB and its microenvironment *in vitro*, several milli- and microfluidic devices have been developed in recent years [25,30,31,32,33,34].

Among the developed models, the hollow-fiber-based millifluidic dynamic *in vitro* (DIV) model from Flocel offers several advantages over static Transwell^®^ models. The most important one is the longevity of the hollow-fiber models with continuous non-invasive monitoring and assessment of BBB integrity during the cultivation through Online-TEER measurements and repeated permeability studies using paracellular markers such as FITC-Dextran 4kDa. BCEC from different cell sources, including the porcine cell line PBMEC/C1-2, primary brain microvascular endothelial cells (HBMECs), and the immortalized human brain microvessel endothelial cell line (hCMEC/D3), were successfully cultivated in the Flocel setup for several weeks to months, allowing long-term studies of the BBB *in vitro* [25,31,35]. In addition, the system allows the co-cultivation of BCEC with BBB-relevant cells, such as astrocytes and pericytes, in two distinctly accessible compartments at physiologically relevant shear rates, with a flow rate and beats per minute adjustable pump system as well.

The first objective of this study was to optimize the culture conditions of hCMEC/D3 cells in the Flocel system for long-term studies over more than one month. The cell line hCMEC/D3 is the most widely used human immortalized BCEC line for modeling the BBB *in vitro*. Previous data revealed that culturing hCMEC/D3 in a hollow-fiber model under dynamic conditions over two weeks led to a reduction of the molar lactate/glucose ratio to a bit above one, indicating a metabolic shift due to shear stress. Since, in these studies, 2% fetal bovine serum was applied in the growth medium, and it is known that hCMEC/D3 cells are still proliferative at this serum concentration, the effects of the reduction of serum concentration in the medium from 5% (standard concentration in the growth medium) to 1% and 0.25% FBS were tested in order to improve the conditions for long-term studies. After defining the optimal culture conditions at 0.25% FBS confirmed by improved barrier integrity, metabolic and gene expression data, as a second objective, a Flocel triple-culture of hCMEC/D3 with human primary astrocytes and pericytes (hAP) was established to investigate its capability for modeling systemic inflammatory processes under physiologically relevant culture conditions (at a shear stress of 5.2 dyne/cm^2^, 0.1 ng/mL and 10 ng/mL cytokine cocktail of TNF-α, IL-1β, INF-γ). In summary, several aims were achieved: optimization of a hollow-fibre BBB *in vitro* model based on hCMEC/D3 cells for long-term cultivation over six weeks for the first time, application of physiologically relevant shear stress of higher than 5 dyne/cm^2^ (which is applied only in a minority of fluidic BBB *in vitro* models), establishment of a triple-culture model of hCMEC/D3 with hAP under these conditions, and usage of this triple-culture model to implement a stable two weeks lasting cytokine response phase, highlighting that physiologically more relevant lower cytokine concentrations in the picogram/mL range were sufficient to achieve sustainable cytokine responses.

## 2. Materials and Methods

### 2.1. Cell Culture

The immortalized human brain capillary cell line hCMEC/D3 (Merck Milipore, Darmstadt, Germany; Ref.: SCC066) was cultivated in T25- or T75-culture flasks (CellStar, Greiner Bio-one, Kremsmünster, Austria; Ref.: 690175 or 658175) coated with 0.5% gelatin (SERVA Electrophoresis GmbH, Heidelberg, Germany; Ref.: 22,151.02) in EBM-2 (Lonza, Basel, Switzerland; CC 3156) supplemented with 5% Fetal Bovine Serum (FBS; Sigma-Aldrich, St. Louis, MO, USA; F9665), 1% penicillin/streptomycin (Biochrom GmbH, Berlin, Germany; A2213), 10 mM HEPES (Sigma-Aldrich, St. Louis, MO, USA; H0887), 5 μg/mL ascorbic acid (Sigma-Aldrich, St. Louis, MO, USA; A4544-25G), and 1 ng/mL hbFGF (Sigma-Aldrich, St. Louis, MO, USA; F0291-25UG). Once a week, hCMEC/D3 were subcultivated by treatment with 0.25% trypsin/EDTA (Biochrom GmbH, Berlin, Germany; L2143) for 3–5 min at 37 °C and replated in a ratio of 1:3 into a freshly gelatin-coated flask. The medium was changed every other day, and HB-FGF was added freshly for each medium exchange.

Human primary astrocytes (hA; Provita AG, Berlin, Germany; SC-1800-5) and human primary pericytes (hP; Provita AG, Berlin, Germany; SC-1200) were cultivated on culture flasks coated with 10 µg/mL P-L-Lysine (P-L-L; ScienCell, Carlsbad, CA, USA; 413) in an astrocyte medium (AM; ScienCell, Carlsbad, CA, USA; 1801) supplemented with 2% FBS (ScienCell, Carlsbad, CA, USA; sc-0010), 1% penicillin/streptomycin (ScienCell, Carlsbad, CA, USA, sc-0503), and 1% astrocyte growth supplement (ScienCell, Carlsbad, CA, USA; sc-1852) or in pericyte medium (PM; ScienCell, Carlsbad, CA, USA; sc-1201) supplemented with 2% FBS (ScienCell, Carlsbad, USA; sc-0010), 1% penicillin/streptomycin (ScienCell, Carlsbad, CA, USA, sc-0503), and 1% pericyte growth supplement (ScienCell, Carlsbad, CA, USA; sc-1252), respectively. hA and hP were subcultivated once a week by treatment with Accutase (Sigma-Aldrich, St. Louis, MO, USA; A6964-100ML) for 2–3 min at 37 °C and were replated at a cell density of 6700 cells/cm^2^ in a freshly P-L-L coated culture flask. The medium was changed every other day. For maintenance, all used cell lines and primary cells were kept in an incubator (Thermo Fisher Scientific, Waltham, MA, USA, HERACell Vios 160i CO_2_ Incubator) at 37 °C in 5% CO_2_ at 95% air atmosphere with 95% humidity.

### 2.2. The DIV Model (Flocel)

**Figure A1:**
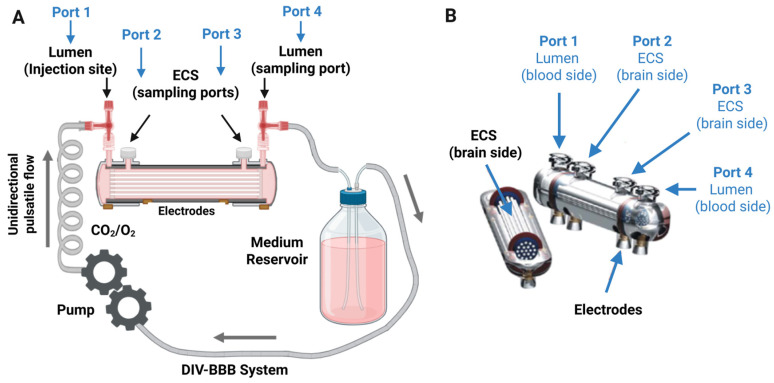
Schematic drawing of the DIV-model set-up (**A**) and the cartridge in detail (**B**). Adapted from Flocel, Inc. (www.Flocel.com (accessed on 13 April 2020)) and Cucullo et al. [35]. Created in BioRender. Neuhaus, W. (2026) https://BioRender.com/em29quk (accessed on 26 June 2026).

The equipment (culture flasks, tubings, peristaltic pump, TEER measuring device) and the cartridges (CAR1010) were purchased from the company Flocel Inc. (Cleveland, OH, USA) (Appendix A, Figure A1). Briefly, a cartridge consisted of 19 polypropylene hollow-fibers (hydrophobic) in a sealed chamber. The fibers and the space surrounding the fibers, the extracapillary space (ECS), were separately accessible by ports. Thus, the fibers represented the blood capillaries, with a diameter of 600 µm each and a total culture surface area of 13.5 cm^2^, whereas the ECS represented the brain parenchyma compartment with a culture surface area of 22.6 cm^2^ on the outside of the plastic hollow fibers. Transcapillary pores (nominal size of 2 µm) in the hollow fibers allowed the exchange of fluids, nutrients and signaling molecules to and from the ECS. The cartridge was connected to a medium reservoir bottle via silicon tubings to build a circular system that allowed the flow of medium within the fibers. Gas exchange (e.g., O_2_ and CO_2_) was performed through silicon tubings. The flow of the medium was controlled by a pulsatile pump (Flocel Quad Pump, QPS5, Flocel Inc., Cleveland, OH, USA) with variable velocities and pulse rates. Transendothelial electrical resistance (TEER) across the cell layer within the fibers was measured in real-time with a TEER measuring device (Flocel Inc., Cleveland, OH, USA) using built-in electrodes within the cartridges. A detailed description of the system was previously published [27,35,36].

**Figure 1:**
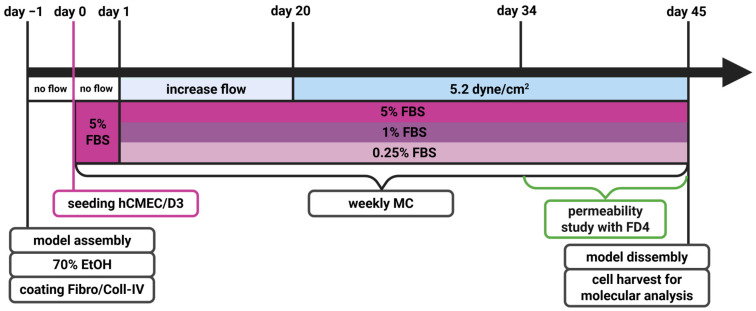
Timeline for the establishment and conduction of the DIV-model mono-culture experiments with hCMEC/D3 cultured with different serum concentrations of 0.25%, 1% or 5% FBS. The term medium change is abbreviated with MC; Fibro/Coll-IV stands for Fibronectin/Collagen-IV.

Before usage, the hollow fibers of the DIV-BBB cartridge were activated with 70% EtOH and washed with sterile Millipore H_2_O to remove the 70% EtOH. The procedure is described in detail in Appendix A.1. The fibers were then coated with 5 mL of a mixture of collagen IV (0.1 mg/mL) and fibronectin (1.0 mg/mL) in sterile PBS or H_2_O overnight at 37 °C. Here, the coating mixture was applied to the lumen and distributed into the ECS to ensure an even coating of the fibers. The procedure for coating was the same as for EtOH activation. On the next day, the coating mixture was removed, and the cartridge was washed two times with sterile PBS or H_2_O, as described in Appendix A.1. After filling the cartridge with a culture medium (supplemented EBM-2 with 5% FBS), hCMEC/D3, with a total cell number of 1.5 × 10^6^ in 5 mL supplemented EBM-2 with 5% FBS, was seeded within the fibers through the luminal ports of the cartridge (details described in Appendix A.2). After the seeding procedure, the ports were sealed with plugs (B. Braun, Maria Enzersdorf, Austria, Ref.: BD394080). Then, the cartridge was rotated 90 degrees every hour for 4 h to allow an even distribution and settlement of the cells within the fibers. After a 24 h attachment period of the hCMEC/D3 without flow, the medium reservoir and the silicon tubings were filled with EBM-2 supplemented with either 5%, 1% or 0.25% FBS. The flow of the medium along the fibers induced a minimal shear stress of 0.37 dyne/cm^2^ (1 mL/min, 40 beats per minute (BPM)) and was increased until 5.2 dyne/cm^2^ (14 mL/min, 75 BPM) was reached over the next 20 days. The total medium volume within the system was 50 mL, whereas the medium reservoir was holding 30 mL. These 30 mL medium in the medium reservoir were changed weekly, and glucose measurements (common glucose strips (OneTouch Select Plus testing strips; LifeScan, Malvern, PA, USA, Ref.: 10963219)) and manual cell counting with a counting chamber (Neubauer improved, Paul Marienfeld GmbH & Co.KG, Lauda-Königshofen, Germany) were performed. Medium samples of the weekly medium change were collected and stored at −20 °C for further metabolite analysis. After five weeks in cultivation, a permeability study with the paracellular marker fluorescein isothiocyanate–dextran (10 μM; FD4; 4 kDa; Sigma-Aldrich, St. Louis, MO, USA; FD4—250MG, 1 mM stock solution of FD4 ultra-filtered with Amicon tubes with a cut-off 3 kDa to separate from residual, free FITC) was performed at 0.37 dyne/cm^2^, whereby samples of the medium reservoir and the ECS were taken every 10 min for 60 min to determine the transport of FD4 across the cell layer within the fibers into the ECS. The permeability coefficient was calculated according to an adapted formula by Levis [31]. Additionally, TEER was measured online repeatedly to determine barrier integrity throughout the experiment. After a cultivation period of up to seven weeks, the model was disassembled, and medium samples were collected and stored at −20 °C til further analysis (Figure 1). The total number of cells collected from the medium reservoir was counted, and cells were harvested from the fibers with 0.25% trypsin/EDTA. The collected cell pellet was lysed in RA-1 buffer (NucleoSpin-RNAII RNA Isolation Kit; Machery-Nagel, Düren, Germany; Ref.: 740.955.250) or RLT buffer (AllPrep DNA\RNA\Protein Mini Kit; Qiagen, Hilden, Germany; Ref.: 80004), both supplemented with 1% β-mercaptoethanol (Sigma-Aldrich, USA; M3148), and stored at −80 °C until RNA isolation.

**Figure 2:**
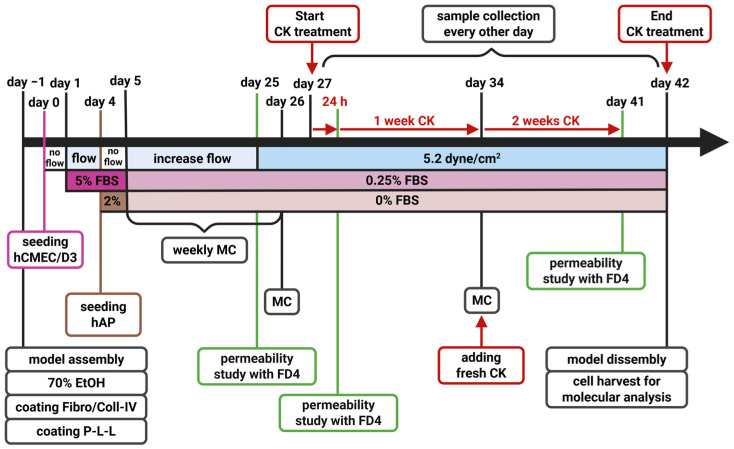
Timeline for the establishment of the DIV model triple culture with hCMEC/D3 in EBM-2 with 0.25% FBS and human primary astrocytes and pericytes (hAP) in serum-free AM and PM (1:1) and experimental cytokine treatment with 0.1 ng/mL or 10 ng/mL TNF-α, IL-1β, and INF-γ or 0.1% BSA as control. The term medium change is abbreviated with MC; Fibro/Coll-IV stands for Fibronectin/Collagen-IV.

For adapting the DIV-model mono-culture to a triple-culture, hCMEC/D3 were co-cultivated with human primary astrocytes and pericytes (hAP) for up to 6 weeks (Figure 2). As described above, cartridges consisting of 19 hollow fibers with a culture surface area of 13.5 cm^2^ were used. The ECS surrounding the hollow fibers allowed the cultivation of human primary astrocytes and pericytes on the extraluminal fiber surface. After activation of the fibers with 70% EtOH (as described above and in the Appendix A.2), the intraluminal surfaces of the hollow fibers were coated with a mixture of collagen IV (0.1 mg/mL) and fibronectin (1.0 mg/mL), whereas the ECS was simultaneously coated with 10 µg/mL poly-L-lysine (P-L-L, ScienCell, Carlsbad, CA, USA; 0413) overnight at 37 °C. The next day, hCMEC/D3 were inoculated into the fibres with a total cell number of 1.5 × 10^6^ cells. After a settling period of four hours with a 90-degree rotation of the cartridge each hour and an overnight incubation at 37 °C without flow, the flow rate was set to 1 mL/min and 40 BPM (0.37 dyne/cm^2^). hCMEC/D3 were cultivated in EBM-2 with 5% FBS for the following four days after inoculation. On day four, the co-culture was established by seeding human primary astrocytes and pericytes with a total cell number of 1.5 × 10^6^ cells (750,000 cells each) in a mixture of AM and PM medium (1:1) with 2% FBS. For an even distribution, the hAP cell suspension was moved through the ECS by gently pushing and pulling the plungers of the syringes mounted to ports 2 and 3 simultaneously five to seven times (Figure A1B), which was followed by a four-hour settling period, during which the cartridge was rotated by 90 degrees hourly. For the seeding procedure and during the settling period, the flow was stopped. After the four-hour settling period, the flow was started again at 1 mL/min, 40 BPM (0.37 dyne/cm^2^). On day six, hCMEC/D3 were re-seeded at a density of 1.5 × 10^6^ in 5 mL supplemented EBM-2 with 5% FBS to replace lost hCMEC/D3 during the process of establishing the triple-culture with hAP. Again, a settling period, with rotation of the cartridge 90 degrees every 30 min, allowed the even attachment of the cells. The next day, the medium in the fibers, the medium reservoir and the silicon tubings were changed to serum-reduced EBM-2 with 0.25% FBS, and the medium in the ECS was changed to a serum-free 1:1 mixture of AM and PM. Between day five and day 26, the medium in the medium reservoir and the ECS was changed weekly, and the flow was increased until a shear stress of 5.2 dyne/cm^2^ was reached. On day 25, a permeability study with FD4 was performed to assess barrier integrity. After the permeability study, the medium was changed to remove residual FD4 from the system. TEER was measured continuously to determine the barrier integrity throughout the experiment. On day 27, the cytokine (CK) treatment was started by adding 0.1 ng/mL or 10 ng/mL of TNF-α (Sigma-Aldrich, USA, Ref.: H8916-10UG), IL-1β (Peprotech, Cranbury, NJ, USA, Ref.: 200-01B), and INF-γ (Peprotech, USA, Ref.: 300-2) diluted in sterile PBS, supplemented with 0.1% BSA (Fraction V; Carl Roth, Karlsruhe-Mühlburg, Germany; Ref.: 8076.3), directly to the 30 mL medium in the medium reservoir (Table S1). As a control, the vehicle, 0.1% BSA (Fraction V; Carl Roth, Ref.: 8076.3), was added to the medium reservoir. A detailed description of the preparation of the CK stock solutions is provided in the Supplementary (Table S1). Medium samples of the lumen/medium reservoir and the ECS were collected after 6h and 24 h of cytokine treatment or vehicle. Additionally, after 24 h of CK treatment, a permeability study with FD4 was performed to determine the immediate effect of the cytokine treatment on BBB integrity. Samples from the lumen and the ECS were collected every other day and stored at −20 °C. Medium change was continued on a weekly basis with the addition of a fresh cytokine cocktail with every medium change. Collected medium samples were used for determining the concentration of secreted proinflammatory cytokines. On day 42, when two weeks of cytokine treatment were reached, a final permeability study with FD4 was performed. hCMEC/D3 were then harvested from the fibers with 5 mL trypsin/EDTA, and hAP were harvested from the ECS with 1 mL Accutase. The collected cell suspensions were centrifuged at 300× *g* for 5 min at room temperature. Cell pellets were lysed in RLT-buffer (AllPrep DNA\RNA\Protein Mini Kit; Qiagen, Germany; Ref.: 80004) supplemented with 1% β-mercaptoethanol (Sigma-Aldrich, USA; M3148) and stored at −80 °C until RNA isolation.

### 2.3. Transwell^®^ Experiments with Cytokine Treatment

hCMEC/D3 were seeded at a cell density of 80,000 cells/cm^2^ in a 300 µL medium onto 24-well inserts (Falcon-BD, pore size: 0.4 µm, PVDF; Corning, Durham, NC, USA; cat.no.: 353095) coated with a mixture of Coll-IV (0.1 mg/mL; Sigma-Aldrich, USA; cat.no.: C5533) and fibronectin (1 mg/mL; Sigma-Aldrich, USA; cat.no.: F1141-5MG) and cultivated in EBM-2 with supplements and 5% FBS for 5 days (apical: 300 µL, basolateral: 900 µL medium). Then, 900 µL EBM-2 with 5% FBS and supplements were added to the basolateral compartment. Human primary astrocytes and pericytes (hAP) were seeded at a cell density of 25,000 cells/cm^2^ each in 500 µL medium onto the wells of a 24-well plate (Falcon-BD, 353504), which were coated with 10 µg/mL P-L-L (ScienCell, USA; cat.no.: 413), and were cultivated in a mixture of 1:1 AM and PM with supplements and 2% FBS for three days. hCMEC/D3 and hAP were cultured separately until the experimental start. One day before starting the cytokine treatment, the culture medium for hCMEC/D3 was changed to a serum-reduced EBM-2 containing supplements and 0.25% FBS, whereas the medium for hAP was changed to a supplemented, serum-free AM and PM mixture (1:1) when confluency of hAP had been reached. On day six of hCMEC/D3 culture, the cytokine treatment was started. For triple-cultures, the inserts containing hCMEC/D3 were put together with wells containing hAP shortly before experimental start.

For the Transwell^®^ experiments, the CK stock solutions of TNF-α (Sigma-Aldrich, USA, Ref.: H8916-10UG), IL-1β (Peprotech, USA, Ref.: 200-01B) and INF-γ (Peprotech, USA, Ref.: 300-2) with a concentration of 1 µg/mL in 0.1% BSA were mixed together in EBM-2 supplemented with 0.25% FBS to generate a working concentration of 0.1 ng/mL or 10 ng/mL CK cocktail (Table S1). Then, 300 µL of these CK cocktails containing either 0.1 ng/mL or 10 ng/mL of TNF-α, IL-1β and INF-γ each was added apically to the hCMEC/D3, whereas 900 µL of EBM-2 with 0.25% FBS, respectively, a mixture of AM and PM (1:1) without the CK cocktail was added basolaterally. As a control, the vehicle, 0.1% BSA (Fraction V; Carl Roth, Ref.: 8076.3), was added to mono- and triple-cultures apically. It should be noted that the CK cocktail was only added apically to the hCMEC/D3 and not basolaterally to the hAP. The supernatant was collected apically and basolaterally after 24 h of cytokine treatment or vehicle and stored at −20 °C. hCMEC/D3, and hAP were harvested after 24 h, lysed in RLT-buffer supplemented with 1% β-mercaptoethanol (Sigma-Aldrich, USA; M3148) and stored at −80 °C for later molecular analysis. For subsequent RNA isolation, cells from three inserts were pooled together during RNA lysis.

### 2.4. RNA Isolation and cDNA Synthesis

RNA isolation of cell pellets lysed in RA-1 buffer was performed with the NucleoSpin-RNAII RNA Isolation Kit (Machery-Nagel, Düren, Germany; Ref.: 740.955.250), whereas RNA isolation of cell pellets lysed in RLT-Buffer was performed with the AllPrep DNA\RNA\Protein Mini Kit (Qiagen, Germany; Ref.: 80004) following the manufacturer’s instructions. The High-Capacity cDNA Reverse Transcription Kit (Applied Biosystems/Thermo Fisher Scientific, Darmstadt, Germany; 4368814) was used to convert the isolated RNA into 250 ng cDNA in 20 µL. Changes in mRNA levels were detected by high-throughput qPCR (Fluidigm^®^), whereby samples were loaded on 96-sample x 96-target Fluidigm^®^ chips. A more detailed description of the analysis process was provided in several recent publications [33,37,38,39,40,41,42]. The analyzed panel consisted of BBB-relevant targets, including tight junction and transporter proteins, as well as inflammation markers, to determine the inflammatory response towards the cytokine treatment or vehicle (Tables S11, S17 and S18). Calculated 2∆Ct-values were normalized to the endogenous house-keeping gene B2M or GAPDH.

### 2.5. Glucose and Lactate Measurement

The concentration (g/L) of glucose and lactate in medium samples collected from the medium reservoir during the weekly medium change of the serum dependency experiments was measured with the Bioprofiler 100 Plus Analyzer (Nova Biomedical, Waltham, MA, USA). The daily glucose consumption and lactate production were calculated according to formulas previously published [31,36,43]. The lactate/glucose ratio was calculated by putting the daily lactate production (µmol/day) in relation to the daily glucose production (µmol/day).

### 2.6. NMR Analysis of Metabolites

Metabolite secretion and consumption by hCMEC/D3 in response to serum reduction were determined through NMR analysis of cell-conditioned medium samples collected from the medium reservoir, compared to acellular medium samples. To precipitate interfering proteins, 350 µL of medium were mixed with 700 µL of methanol (precooled at −80 °C), kept at −20 °C for 30 min and centrifuged at 13,000× *g* for 20 min. Then, 1000 µL of the supernatant was transferred to a new microtube and dried under vacuum in a Speedvac concentrator (model 5301, Eppendorf, Hamburg, Germany). For NMR analysis, dried samples were reconstituted in 600 µL of deuterated PBS (pH 7.4, 100 mM, containing 0.1 mM TSP-d4), and 550 µL were transferred into 5 mm NMR tubes. Samples were analyzed on a Bruker Avance III HD 500 spectrometer (Bruker BioSpin GmbH & Co. KG, Ettlingen, Germany, University of Aveiro, Portuguese NMR Network) operating at 500.13 MHz for 1H observation, at 298 K. Standard 1D 1H NMR spectra (pulse program ‘noesypr1d’) were acquired with a 7002.8 Hz spectral width, 32 k data points, a 2 s relaxation delay, and 512 scans. Spectral processing in TopSpin 4.0.9 (Bruker BioSpin, Rheinstetten, Germany) comprised cosine multiplication (ssb 2), zero-filling to 64 k data points, manual phasing and baseline correction, and calibration to the TSP-d4 signal (δ 0 ppm). For quantitative measurement of the metabolic variations, selected signals representative of individual metabolites were integrated with Amix-Viewer 3.9.15 (Bruker Biospin, Rheinstetten, Germany). Signal areas were then used to calculate the variations in cell-conditioned media relative to the acellular media. Changes in the metabolic activity of cells cultivated in DIV-model mono-cultures with varying serum concentrations (5% FBS, 1% FBS and 0.25% FBS) were determined by comparing the metabolite signal areas in the cell-conditioned medium to those in the corresponding acellular fresh EBM-2 medium containing either 5%, 1% or 0.25% FBS [44].

### 2.7. Quantibody Cytokine Array

To analyze secreted cytokines from endothelial cells, as well as from human primary astrocytes and pericytes as a response to the CK treatment, a multiplexed sandwich ELISA-based quantitative array “Quantibody” (RayBiotech, Peachtree Corners, GA, USA, Ref.: QAH-INF-1) was performed according to the manufacturer’s instructions. Samples of the medium reservoir, the ECS and the apical and basolateral samples from Transwells^®^ were analyzed. Samples were diluted 1:5 (40 µL + 160 µL) in serum-free EBM-2 without supplements, followed by a 1:2 dilution (100 µL +100 µL) with Quantibody^®^ Sample Diluent. Glass slides were analyzed with a TECAN-PowerScanner (Tecan, Zurich, Switzerland, Ref.: 1311006393; excitation wavelength 532 nm, 10 µm resolution, PMT gain = 0). Images were manually gridded with the software “GenePixPro, Version 7.3”. After image preparation, the results were read out with an analysis file provided by RayBiotech, and the results were multiplied by 10 to compensate for the sample dilution.

### 2.8. Statistics

Statistical analysis was performed by One-Way ANOVA with TSD testing, with Dunn’s method or Holm–Sidak for post hoc testing, as well as by Two-Way ANOVA with all pairwise multiple comparison Holm–Sidak in case of non-normality distribution or non-equal variances using SigmaPlot 14. Student’s t-test was performed to determine significant metabolic changes of the cellular medium compared to the acellular medium. Hierarchical cluster analysis was performed with the statistical software “R” (version 4.0.4; R Foundation for Statistical Computing, Vienna, Austria; URL: http://www.R-project.org/) with the R package “pheatmap” [45]. Graphic visualizations of data were prepared with R, and for axis breaks, the R package “ggbreak” was used [46]. Biorender.com was used for figure preparation.

## 3. Results

### 3.1. Serum Reduction Led to Decreased Cell Proliferation, Induced Aerobic Metabolism and Improved Barrier Integrity in the hCMEC/D3 DIV Model

When exposed to an excessive amount of nutrients, the immortalized human brain capillary cell line hCMEC/D3 is prone to uncontrolled proliferation, which could cause cellular overgrowth, preventing the formation of a hCMEC/D3 monolayer within the DIV model and, further on, leading to a reduced barrier integrity [47,48,49].

To prevent excessive proliferation of hCMEC/D3 cells in the DIV model, serum concentrations were reduced from the standard 5% FBS to 1% FBS or 0.25% FBS from day 1 after seeding. Cell proliferation and metabolic activity were monitored throughout the experiment duration for up to seven weeks. Lower serum concentrations reduced cell expansion, as reflected by decreased numbers of cells recovered during weekly medium changes and lower endpoint RNA yields. The strongest effect was observed at 0.25% FBS, which markedly suppressed cell proliferation compared with 1% FBS and 5% FBS cultures (Figure S2; Tables S2–S4).

**Figure 3:**
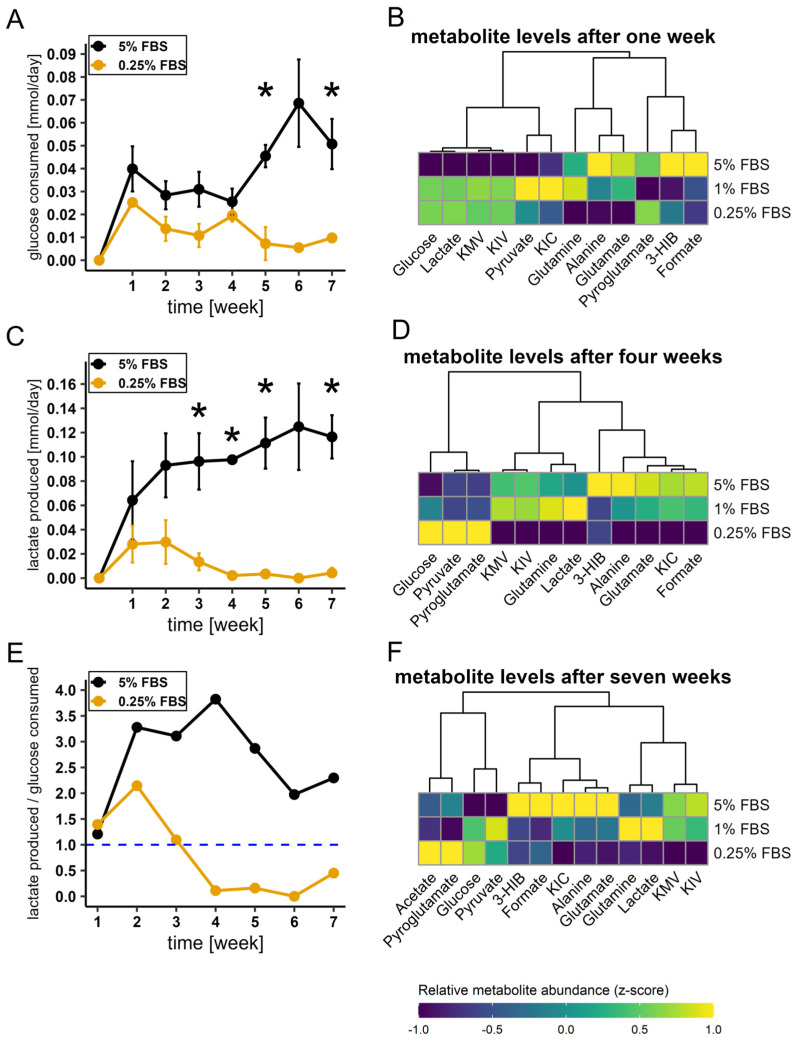
Metabolic analysis of hCMEC/D3 cultivated in the DIV model as mono-culture with 5% FBS, 1% FBS or 0.25% FBS. (**A**) The glucose consumption (mmol/day) of hCMEC/D3 was significantly higher in cultures with 5% FBS compared to cultures with 0.25% FBS in week 5 and week 7 (n = 2–4, N = 3–4). (**C**) Lactate production (mmol/day) in Flocel mono-cultures with 5% FBS was significantly increased compared to mono-cultures with 0.25% FBS for weeks 3, 4, 5 and 7 (n = 2–4, N = 3–4). (**E**) The ratio of lactate produced over glucose consumed per day over a cultivation period of seven weeks revealed possible metabolic differences in hCMEC/D3 cultivated in 5% FBS to 0.25% FBS. The blue dashed line (lactate/glucose ratio = 1) marks the threshold between predominantly glycolytic (ratio > 1) and oxidative (ratio < 1) glucose metabolism (n = 3–4, N = 3–4). The heatmaps show significant changes in the secretion or consumption of metabolites in hCMEC/D3 cultivated in 5%, 1% or 0.25% FBS compared to acellular medium after a cultivation period of one week (**B**), four weeks (**D**) or seven weeks (**F**). Colors represent column-scaled metabolite abundances (z-scores), where positive values indicate higher and negative values indicate lower abundance relative to the mean abundance of each metabolite across all samples. Samples were hierarchically clustered, and a common color scale (−1 to 1) was used across all heatmaps (n = 2–3, N = 2–3). Data are presented as mean ± SEM. One-way ANOVA with Tukey’s HSD for post hoc testing; *p* < 0.05, * 5% FBS significant against 0.25% FBS.

To determine if the serum-dependent reduction in proliferation might be linked to an altered metabolism of hCMEC/D3, the consumption of glucose and the production of lactate in hCMEC/D3 cultivated with the standard FBS concentration of 5% was compared to those of hCMEC/D3 cultivated with the lowest FBS concentration of 0.25%. Indeed, a significantly reduced average glucose consumption (0.01 ± 0.004 mmol/day) and lactate production (0.02 ± 0.003 mmol/day) of hCMEC/D3 cultivated with 0.25% FBS was determined compared to the average glucose consumption (0.04 ± 0.01 mmol/day) and lactate production (0.10 ± 0.01 mmol/day) over time of the hCMEC/D3 cultivated with 5% FBS (Figure 3A,C; Table S5).

The ratio of lactate production to glucose consumption can provide insights into the balance between glycolytic and oxidative metabolism. A higher lactate/glucose ratio (>1) suggests increased reliance on glycolysis with lactate production, whereas a lower ratio (<1) indicates greater glucose oxidation via the TCA cycle and oxidative phosphorylation [35,50,51]. A significant reduction in the average lactate/glucose ratio from 2.53 ± 0.33 (in 5% FBS) to 0.75 ± 0.28 (in 0.25% FBS) suggested a metabolic shift away from glycolysis towards a greater reliance on oxidative metabolism under lower serum concentrations over the seven weeks of cultivation (Figure 3E; Table S5).

To further investigate metabolic changes in hCMEC/D3 cultivated under different serum concentrations (5%, 1% or 0.25% FBS), an in-depth analysis of metabolite consumption and secretion was performed using NMR spectroscopy (Figure 3B,D,F; Tables S6–S8) [44]. In general, the metabolites pyruvate, glucose, and glutamine were consumed (i.e., showed decreased levels compared to the corresponding cell-free medium), while acetate, alanine, formate, glutamate, pyroglutamate, glycine, 3-hydroxyisobutyrate (3-HIB), α-ketoisocaproate (KIC), α-ketoisovalerate (KIV), α-keto-β-methylvalerate (KMV), and lactate were secreted. A closer analysis of the time-dependent response revealed that, after one week of cultivation (Figure 3B and Figure S3A, Table S6), glucose consumption decreased significantly under lower serum concentrations. Moreover, cells in 1% and/or 0.25% FBS showed significantly lower secretion of alanine, formate, glutamate, and 3-HIB compared to cells in 5% FBS. These serum-dependent differences persisted after four and seven weeks of cultivation (Figure 3D,F and Figure S3B,C, Tables S7 and S8, respectively). Additionally, at four weeks, cells in 0.25% FBS consumed less pyruvate and secreted lower amounts of branched chain α-keto acids KMV and KIV compared to 1% and 5% FBS culture conditions. Lactate secretion also showed the lowest magnitude at 0.25% FBS. At seven weeks, cells additionally showed a difference in glutamine consumption (lower at 1% FBS) and in the secretion of pyroglutamate (higher at 0.25% FBS) and KIV (higher at 5% FBS).

In summary, the reduced proliferation of hCMEC/D3 cultured with 0.25% FBS was linked to significant metabolic alterations, including a shift towards aerobic respiration, as indicated by the molar lactate/glucose ratio lower than one, and changes in amino acids and TCA cycle-related metabolites.

### 3.2. Serum Reduction Increased Barrier Integrity

**Figure 4:**
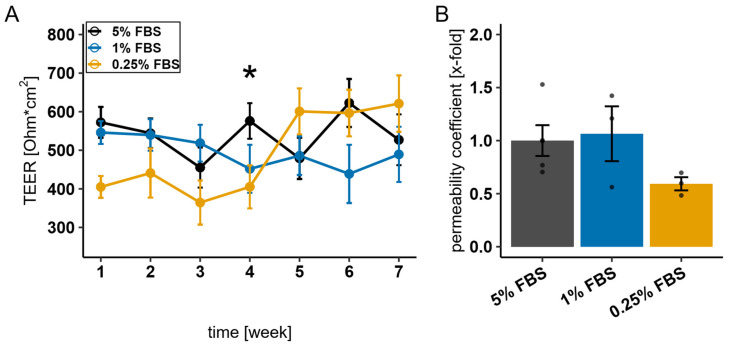
Assessment of barrier integrity of hCMEC/D3 cultivated in the DIV model as mono-culture in 5% FBS, 1% FBS or 0.25% FBS. (**A**) TEER of monocultures in Ohm*cm^2^ comparing hCMEC/D3 cultivated in 5%, 1% and 0.25% FBS for up to seven weeks (n = 6–56, N = 3–4). (**B**) Permeability coefficient of paracellular marker FD4 across hCMEC/D3 layers assessed after cultivation with 5%, 1% or 0.25% FBS at the end of the experiments (n = 3–5, N = 3–5). Data are presented as mean ± SEM. One-way ANOVA with Dunn’s Method for post hoc testing. *p* < 0.05, * significant against 5% FBS.

To determine if the cultivation with different serum concentrations affects the barrier properties of hCMEC/D3 cultivated in the DIV model, the barrier integrity was assessed by transendothelial electrical resistance (TEER) measurements over time and by permeability studies with the paracellular marker FITC-Dextran 4kDa (FD4) across the cell layer formed by hCMEC/D3 within the fibers to the extracapillary space (ECS) of the DIV cartridge (Figure 4). Cultivation of hCMEC/D3 under reduced serum conditions did not impair TEER values over time, although a significant difference in TEER between hCMEC/D3 cultured with 5% FBS and hCMEC/D3 cultured in 0.25% FBS was determined after week four (576 ± 45 Ohm*cm^2^ against 304 ± 30 Ohm*cm^2^). Interestingly, BBB integrity seemed to increase slightly in hCMEC/D3 cultured with 0.25% FBS after four weeks, when the targeted shear stress of 5.2 dyne/cm^2^ had been reached (Figure 4A; Table S9).

This slight increase in BBB integrity of the hCMEC/D3 cell layers cultured with 0.25% FBS was further supported by a decrease in the permeability coefficient of the paracellular marker FD4 for cultures with 0.25% FBS (7.10 ± 0.60 µm/min (0.59 ± 0.05 fold)) compared to the cultures with 5% FBS (11.97 ± 1.55 µm/min (1.00 ± 0.13 fold), although the difference was not statistically significant (Figure 4B; Table S10).

### 3.3. Serum Reduction Caused Changes in the mRNA Levels of BBB Markers

**Figure 5:**
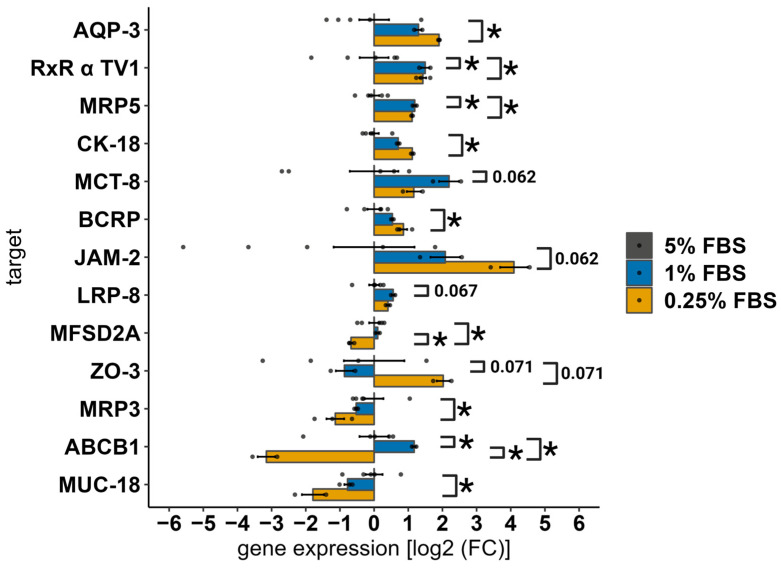
Analysis of gene expression changes in hCMC/D3 cultivated in the DIV model as mono-culture for up to seven weeks at different serum concentrations of 5% FBS, 1% FBS or 0.25% FBS. Significant changes were determined on the mRNA level as fold changes for targets, including tight junction and transporter proteins, when comparing hCMEC/D3 cultivated in 5% FBS, 1% FBS or 0.25% FBS. Values were normalized to the housekeeping gene B2M and then put into relation to values of mono-cultures cultivated in 5% FBS. Targets that showed significance are presented in log2(FC) scale to ease graphic display (n = 2–5; N = 2–5). Data are presented as mean ± SEM. One-way ANOVA with Dunn’s Method for post hoc testing; *p* < 0.05, * significant against 5% FBS or 1% FBS.

In addition to the moderate effects on BBB integrity, significant changes in mRNA expression were observed in hCMEC/D3 corresponding to the degree of serum reduction (5% to 1% to 0.25% FBS) determined by high-throughput qPCR assessing the gene expression of BBB-relevant targets, including tight junction (TJ) and ABC transporter proteins. Data were normalized to hCMEC/D3 cells cultivated in the DIV model as a mono-culture in 5% FBS (Figure 5; Table S11). Hierarchical clustering was performed to reveal target clusters with similar regulation after serum reduction to 1% or 0.25% FBS (Figure S4). Significant upregulations in mRNA levels of hCMEC/D3 cultured with 0.25% FBS compared to mono-cultures in 5% FBS were determined for the targets AQP-3 (3.73 ± 0.02 fold), BCRP (1.81 ± 0.14 fold), CK-18 (1.63 ± 0.03 fold), JAM-2 (17.06 ± 4.53 fold, trending), MRP5 (2.16 ± 0.02 fold), and RXRα TV1 (2.69 ± 0.19 fold), whereby MRP5 (2.28 ± 0.07 fold) and RXRα TV1 (2.82 ± 0.23 fold) were also upregulated in hCMEC/D3 cultured with 1% FBS. LRP-8 (1.47 ± 0.04 fold) and MCT-8 (4.57 ± 0.89 fold) showed a trend towards upregulation only in hCMEC/D3 cultured with 1% FBS. The targets MFSD2A (0.63 ± 0.02 fold), MRP3 (0.46 ± 0.08 fold) and MUC-18 (0.29 ± 0.06 fold) were significantly downregulated in hCMEC/D3 cultured with 0.25% FBS. Interestingly, ABCB1 was significantly upregulated in mono-cultures with 1% FBS (2.25 ± 0.07 fold) but significantly downregulated in mono-cultures with 0.25% FBS (0.11 ± 0.02 fold), whereas ZO-3 showed a trend towards downregulation in mono-cultures in 1% FBS (0.55 ± 0.09 fold) and a trend towards upregulation in mono-cultures in 0.25% FBS (4.06 ± 0.53 fold). In summary, the gene expression analysis revealed serum-dependent effects and confirmed a significant expression of BBB markers in hCMEC/D3 cultured with 0.25% FBS.

All in all, the data supported the use of 0.25% FBS for further studies. Since proliferation was restricted, metabolism shifted towards aerobic respiration over several weeks. It prevented uncontrolled cell growth within the reactor, and barrier integrity was increased, supported by the expression of BBB-relevant tightness markers [35].

### 3.4. Cytokine Treatment for 14 Days Did Not Lead to Severe Barrier Breakdown

The barrier properties of BCEC are highly affected by microenvironmental cells such as astrocytes and pericytes [7,11]. Thus, to further refine the DIV-model set-up with hCMEC/D3 cultivated in 0.25% FBS, a triple culture of hCMEC/D3 cultivated within the fibers and a 1:1 mixture of human primary astrocytes and pericytes (hAP) cultivated in the ECS of the DIV cartridge, surrounding the fibers, was established, allowing the optimization of a more physiologically relevant DIV model of the human BBB. The applicability of this optimized DIV model as a disease model for systemic inflammatory processes was assessed. After four weeks of cultivation, when the targeted level of shear stress had been reached (5.2 dyne/cm^2^), a cytokine cocktail (CK) containing 0.1 ng/mL or 10 ng/mL of TNF-α, IL-1β and INF-γ (diluted in PBS with 0.1% BSA) was added to the medium reservoir, and the cells were treated with the CK cocktails for up to two consecutive weeks.

**Figure 6:**
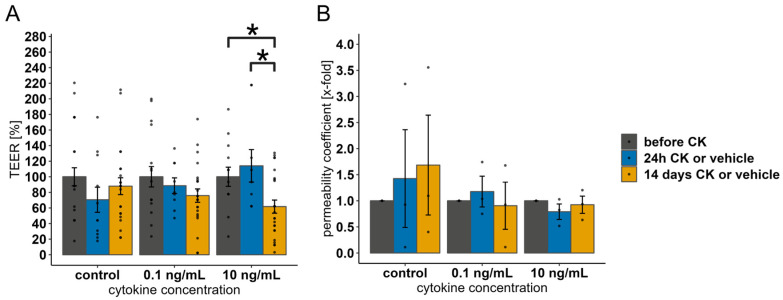
Analysis of barrier integrity of hCMEC/D3 triple culture with hAP in the DIV model before and during cytokine treatment with 0.1 ng/mL or 10 ng/mL of TNF-α, IL-1β or INF-γ each. (**A**) TEER-values were measured before cytokine treatment with 0.1 ng/mL or 10 ng/mL TNF-α, IL-1β and INF-γ (CK) or 0.1% BSA as vehicle after 24 h after start of CK treatment and after 14 days of CK treatment. After subtraction of blank, TEER values were normalized to TEER values generated before CK treatment [%] (n = 9–25, N = 2–3). (**B**) Permeability coefficients of paracellular marker FD4 across hCMEC/D3 layers from the fibers into the ECS after 24 h or 14 days CK treatment, compared to the permeability coefficient before CK treatment. Calculated Pe-values for 24 h CK treatment and 14 days treatment or vehicle were put into relation to Pe-values before CK treatment (x-fold) (n = 2–3, N = 2–3). Data are presented as mean ± SEM. * significant with *p* < 0.05; Two-way ANOVA with all pairwise multiple comparisons according to the Holm–Sidak method was used in case of non-normality distribution or non-equal variances of analyzed data.

TEER and FD4 permeability of the control triple-cultures remained stable over the 14 days of treatment. Similar to the control (0.1% BSA diluted in PBS added), triple-cultures treated with a 0.1 ng/mL CK cocktail revealed a constant TEER and PC throughout the experiment with an average TEER of 509 ± 54 Ohm*cm^2^ (89 ± 10%) and an average PC of 36.18 ± 13.91 µm/min (1.33 ± 0.21-fold) after 24 h CK treatment, as well as an average TEER of 436 ± 48 Ohm*cm^2^ (76 ± 8%) and an average PC of 31.91 ± 14.89 µm/min (1.17 ± 0.17-fold) after 14 days of CK treatment compared to an average TEER of 574 ± 73 Ohm*cm^2^ (100 ± 13%) and an average PC of 27.17 ± 6.91 µm/min (1.00 ± 0.32-fold) before CK treatment. Significant regulations in TEER were determined only in triple-cultures after 10 ng/mL of CK treatment, with an average TEER of 868 ± 102 Ohm*cm^2^ (100 ± 12%) before CK treatment that seemed to be slightly increased to 990 ± 171 Ohm*cm^2^ (114 ± 20%) after 24 h CK treatment, followed by a significant decrease to 536 ± 71 Ohm*cm^2^ (61 ± 8%) after 14 days CK treatment. Again, no changes in FD4 permeability were determined for the triple-cultures treated with a 10 ng/mL CK cocktail after 24 h or 14 days of CK treatment compared to permeability before CK treatment (Figure 6A,B; Tables S12 and S13).

In summary, the treatment with a 0.1 ng/mL and 10 ng/mL CK cocktail caused minor changes in the BBB integrity of hCMEC/D3 triple-cultures in the DIV model.

### 3.5. Systemic Inflammatory Conditions Caused Sustained and Concentration-Dependent Secretion of Proinflammatory Cytokines

To determine whether physiologically relevant CK concentrations are sufficient to induce the secretion of proinflammatory cytokines by BCEC, astrocytes and pericytes *in vitro*, medium samples were collected from hCMEC/D3 co-cultured with hAP following treatment with either a 0.1 ng/mL or 10 ng/mL CK cocktail. As markers for the proinflammatory response, the secretion levels of the proinflammatory cytokines IL-6, IL-8 and MCP-1 were quantified using the Quantibody multiplex array. Preliminary experiments in Transwell^®^ models, consisting of apically seeded hCMEC/D3 co-cultured with basolateral hAP, exposed to 0.1 ng/mL or 10 ng/mL CK cocktail added apically, demonstrated a concentration-dependent increase in IL-6, IL-8 and MCP-1 secretion after 24 h apically and basolaterally (Figure S5; Table S14). Especially, the additional increase in released cytokines in the basolateral compartment induced by apically administered cytokines confirmed the relevance of the basolaterally seeded hAP.

**Figure 7:**
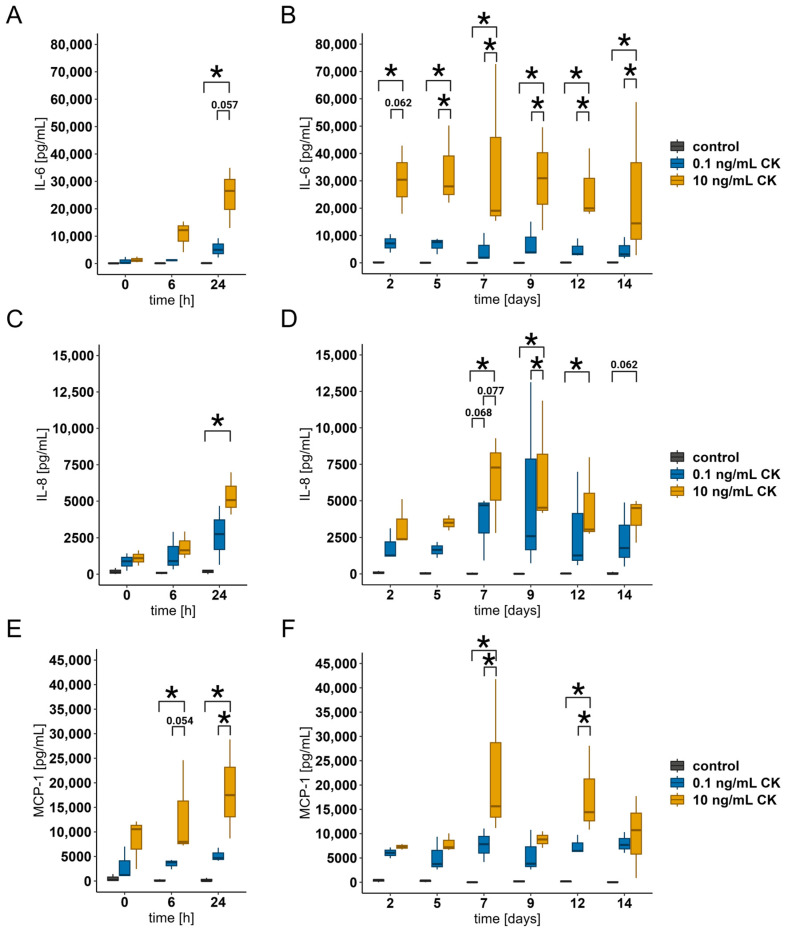
Treatment with 0.1 ng/mL or 10 ng/mL TNF-α, IL-1β and INFγ (CK) led to the secretion of proinflammatory cytokines IL-6, IL-8 and MCP-1 within 6 h and 24 h (**A**,**C**,**E**) and within the following 14 days of CK treatment (**B**,**D**,**F**) in hCMEC/D3 cultivated within the fibers of the DIV cartridge. It should be noted that medium changes, including fresh CKs, were performed after the permeability study after 24 h CK treatment, as well as on day 7 after the start of CK treatment (see Figure 2) (n = 3, N = 3). Data are presented as mean ± SEM. Two-way ANOVA, all pairwise multiple comparison Holm–Sidak was used in case of non-normality distribution or non-equal variances of data; *p* < 0.05, * significant against 0.1 ng/mL CK or 10 ng/mL CK.

To evaluate early responses in the triple-culture DIV model, samples from both the fiber compartment (“blood side”) and the extracapillary space (ECS; “brain side”) were compared after 6 h and 24 h of treatment to investigate the sustainability of the CK treatments. Samples were collected every second day until day 14 and compared with time-matched control cultures receiving 0.1% BSA (Figure 7 and Figure S6; Tables S15 and S16).

CK treatment induced a concentration-dependent increase in the secretion of IL-6, IL-8, and MCP-1 throughout the experimental period (Figure 7). Minor fluctuations in cytokine levels were observed following the weekly medium replacement and re-administration of CKs. In the fiber compartment, the IL-6 concentrations were significantly elevated following treatment with a 10 ng/mL CK cocktail at all assessed time points from 24 h to day 14 compared with controls. Treatment with a 0.1 ng/mL CK cocktail also significantly increased IL-6 secretion, although to a lesser extent than 10 ng/mL CK (Figure 7A,B; Table S15). Similarly, IL-6 concentrations in the ECS samples were significantly increased after treatment with 10 ng/mL CK cocktail at multiple time points, with significant differences between the 0.1 ng/mL and 10 ng/mL treatment groups observed after 24 h, 5 days, and 9 days of exposure, confirming the effects of luminal added CKs on abluminal CK concentrations (Figure S6; Table S16). Comparable concentration-dependent responses were observed for IL-8 and MCP-1.

Overall, luminal exposure to the CK cocktail at 0.1 ng/mL and 10 ng/mL significantly enhanced IL-6, IL-8, and MCP-1 secretion in the DIV-model triple cultures in the luminal, as well as in the abluminal, compartment. Elevated cytokine levels were maintained throughout the 14-day treatment period, indicating that a sustained proinflammatory response can be induced and maintained under dynamic culture conditions.

### 3.6. Systemic Inflammatory Conditions Induced Changes in the mRNA Expression Pattern of hCMEC/D3 After 14 Days

**Figure 8:**
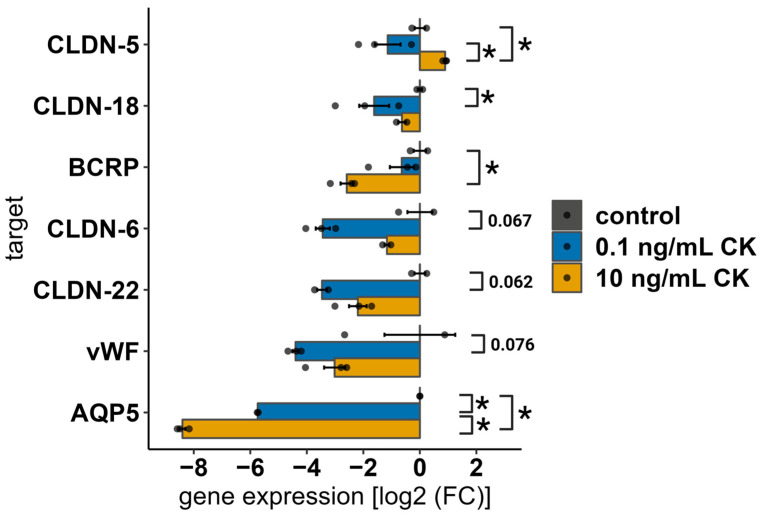
Analysis of mRNA level changes after 14 days CK treatment with 0.1 ng/mL or 10 ng/mL CK cocktail. Treatment with 0.1 ng/mL or 10 ng/mL TNF-α, IL-1β and INF-γ (CK) or 0.1% BSA as control for 14 days led to changes at the mRNA levels of several tight junction and transporter proteins relevant for BBB integrity in hCMEC/D3 cultured in the fibers of the DIV cartridges measured with high-throughput qPCR. Values were normalized to the housekeeping gene GAPDH and then put into relation to values of the cultures treated with 0.1% BSA (control). Trending or significantly regulated targets are presented and are shown in log2(FC) scale for easier graphic display (n = 2–3, N = 2–3). Data are presented as mean ± SEM. One-way ANOVA with Dunn’s Method for post hoc testing; *p* < 0.05, * significant against control or 0.1 ng/mL CK cocktail.

To determine if CK treatment with 0.1 ng/mL or 10 ng/mL TNF-α, IL-1β and INF-γ not only affected the secretion of proinflammatory cytokines but also caused changes in the gene expression of hCMEC/D3, high-throughput qPCR with a BBB-specific target panel was performed. Preliminary studies with hCMEC/D3 co-cultured with hAP in Transwell^®^ models with apical CK treatment for 24 h revealed significant changes in the mRNA levels of several targets. Applying hierarchical clustering suggested a regulatory network dependent on CK concentration (Figures S7 and S8; Table S17). Results of the extended CK treatments (14 days) on the gene expression levels of hCMEC/D3 cultivated in the triple-culture DIV model are shown in Figure 8.

Several significantly regulated targets were determined, and hierarchical clustering revealed groups of targets that show similar regulations in response to CK treatment after 14 days, confirming CK concentration-dependent effects (Figure S9; Table S18). A trend or a significant downregulation in the mRNA levels of hCMEC/D3 treated with 0.1 ng/mL CK cocktail for 14 days compared to the control was determined for CLDN-6 (0.09 ± 0.02 fold, *p* = 0.067), CLDN-18 (0.33 ± 0.11 fold), CLDN-22 (0.09 ± 0.01 fold, *p* = 0.062) and vWF (0.05 ± 0.004, *p* = 0.076), whereas BCRP was significantly decreased in hCMEC/D3 treated with a 10 ng/mL CK cocktail (0.17 ± 0.02 fold) compared to the control. The gene expression of AQP-5 was significantly decreased in hCMEC/D3 treated with a 0.1 ng/mL (0.02 ± 0.0002 fold) and 10 ng/mL CK cocktail (0.003 ± 0.0002 fold) compared to control, with a significant decrease in expression level in accordance with the increase in CK concentration. Interestingly, the expression level of CLDN-5 decreased after treatment with 0.1 ng/mL CK (0.45 ± 0.15 fold) but significantly increased after treatment with 10 ng/mL CK cocktail (1.86 ± 0.04 fold). These data show that, already at a low CK concentration of 0.1 ng/mL, CK molecular changes can be induced and persist for 14 days of CK treatment, though some targets seemed to be regulated differently depending on the CK concentration. Mainly, tight junction proteins seemed to be affected by the prolonged CK treatment.

In summary, hCMEC/D3 cultivated in the DIV model as a triple-culture with hAP were responsive to both lower (0.1 ng/mL) and higher (10 ng/mL) concentrations of TNF-α, IL-1β and IFN-γ. Under dynamic culture conditions, proinflammatory responses in hCMEC/D3 and hAP were sustained at relatively stable levels for up to 14 days, highlighting the suitability of this model for longer-term studies of systemic inflammatory responses. Although only minor changes in paracellular BBB integrity were observed following CK treatment with 10 ng/mL TNF-α, IL-1β and IFN-γ, the secretion of proinflammatory cytokines IL-6, IL-8 and MCP-1 increased significantly in a CK concentration-dependent manner and remained elevated for 14 days. MRNA level alterations associated with CK treatment were also detected after 14 days of CK treatment, indicating that, even at low concentrations of 0.1 ng/mL CKs without paracellular BBB breakdown, cytokines can affect hCMEC/D3 and may alter the inter- and intracellular processes of brain endothelial cells.

## 4. Discussion

The study had two main goals. First, the culture conditions of the human immortalized brain endothelial cell line hCMEC/D3 had to be optimized to allow long-term cultivation under a shear stress of 5.2 dyne/cm^2^ in a DIV-hollow fiber model longer than four weeks. Since most of the established fluidic BBB *in vitro* models were not reported for longer cultivation times than three weeks, this would have been an exceptional improvement for long-term studies at physiologically relevant shear rates [52,53,54,55]. Second, using these models for longer-term studies under inflammatory-inducing conditions by the addition of physiologically relevant CK concentrations in the pg/mL range would have been a further significant improvement, since most BBB *in vitro* studies investigated CK treatments during the short-term, up to 72 h, although some of them applied CKs in the pg/mL range [56,57,58,59]. To achieve the first goal, the effects of serum reduction from 5% FBS to 1% FBS or to 0.25% FBS on BBB integrity, cell proliferation, metabolism, and gene expression of hCMEC/D3 were assessed in the DIV hollow fiber model over several weeks. hCMEC/D3 is a reliable, stable and easy-to-handle cell line commonly used for *in vitro* modeling of the BBB [41,60,61,62]. Static culture conditions, such as Transwells^®^, are well-suited for short-term studies but lack the capacity to maintain the continuous barrier stability required for long-term studies. A way to enable long-term studies with hCMEC/D3 is to use dynamic culture systems with organotypic features, such as flow along the endothelial cell layers. However, some challenges persist. For instance, in this study, strong proliferation of hCMEC/D3 was observed in the DIV model when cells were cultured in a standard medium containing 5% FBS. This raised the question of whether a high serum concentration caused hCMEC/D3 to overgrow, forming multilayers instead of continuous monolayers. One approach to overcome excessive proliferation is to reduce FBS concentration in the culture medium, as FBS contains an undefined mix of growth factors, nutrients, and vitamins that can influence cell growth [49]. In concordance with this, previous studies have shown that culturing brain endothelial cells in a serum-reduced or serum-free medium can lead to improved barrier properties [47,48,49]. Recently, the establishment of a dynamic co-culture model of hCMEC/D3 with perivascular stem cells, as well as with blood mononuclear cells, was enabled by cultivation in a serum-free medium [63,64].

In the current study, a significant effect of serum reduction on the paracellular barrier properties of hCMEC/D3 cultivated as a mono-culture in the DIV model with 0.25% FBS could not be consistently observed over time, although there seemed to be a trend towards increased TEER and decreased permeability of the paracellular marker FD4 in hCMEC/D3 cultured with 0.25% FBS (Figure 4).

The reduced proliferation of hCMEC/D3 cells at lower FBS concentrations resulted likely from alterations in cellular metabolism. This hypothesis was supported by the found lactate/glucose ratio below one and changes in the secretion and consumption of metabolites involved in central carbon metabolism, pointing towards a metabolic shift from anaerobic to aerobic respiration in hCMEC/D3 cultured with 0.25% FBS (Figure 3; Tables S7–S9). After four to seven weeks of cultivation, hCMEC/D3 in 0.25% FBS showed marked alterations in metabolite levels—namely alanine, formate, glucose, glutamate, glutamine, 3-HIB, KIC, KIV, KMV, lactate, pyroglutamate, and pyruvate—compared to cells cultured in 1% or 5% FBS. Many of these metabolites are directly linked to bioenergetic and biosynthetic pathways that converge on the tricarboxylic acid (TCA) cycle, where they function as carbon sources or intermediates [12,13,14,15,16,17]. In endothelial cells, the primary sources of carbon for the TCA cycle include glucose (via conversion of pyruvate to acetyl-CoA), glutamine (via conversion to glutamate and subsequently to α-ketoglutarate), and fatty acids (via β-oxidation, yielding acetyl-CoA and supporting nucleotide synthesis) [14]. Among these, glutamine is a particularly important substrate; it is converted to glutamate by glutaminase and then to α-ketoglutarate, contributing directly to TCA-cycle anaplerosis. The observed increase in pyroglutamate secretion, together with reduced extracellular glutamate levels under 0.25% FBS, suggests an alteration of glutamine/glutamate metabolism. Pyroglutamate (5-oxoproline) is an intermediate of the γ-glutamyl and glutathione cycles and may reflect changes in glutamate utilization, glutathione turnover, or cellular redox metabolism [65,66,67,68,69]. Thus, these findings are compatible with increased utilization of glutamine-derived metabolites under low-serum conditions, although they do not directly demonstrate increased glutaminolytic flux into the TCA cycle.

In addition, reduced extracellular levels of the branched-chain α-keto acids (BCKAs) KIC, KMV, and KIV, as well as 3-HIB in 0.25% FBS, may indicate altered branched-chain amino acid (BCAAs: leucine, isoleucine, and valine) metabolism. These BCKAs are derived from the transamination of the BCAAs via branched-chain aminotransferases (BCATs) [70,71,72]. Following transamination, BCAA-derived carbon can contribute to mitochondrial metabolism through the formation of acetyl-CoA and/or propionyl-CoA, the latter being converted to succinyl-CoA. The reduced extracellular levels of these metabolites may reflect increased intracellular utilization to replenish TCA intermediates. However, altered formation or secretion of these metabolites cannot be excluded based on extracellular measurements alone.

Changes in acetate, alanine, glycine, and lysine further indicate an adaptation of amino acid and central carbon metabolism, driven by the reduced presence of serum-derived growth factors and nutrients under low-serum conditions [14,73,74,75,76,77]. Collectively, these metabolite patterns, together with the reduced molar lactate/glucose ratio, argue against predominant conversion of glucose to lactate and are consistent with reduced lactate-producing glycolysis and increased utilization of alternative mitochondrial substrates. Nevertheless, because extracellular metabolite concentrations represent net uptake and secretion, these data cannot conclusively establish increased TCA-cycle flux or oxidative phosphorylation.

In summary, the metabolic profile of hCMEC/D3 cells cultured in 0.25% FBS supports a less glycolytic and potentially more oxidative or metabolically quiescent phenotype. This interpretation is consistent with reduced proliferation and maintained or improved barrier function. Direct measurements of OCR (oxygen consumption rate), ECAR (extracellular acidification rate), mitochondrial function, or isotope-resolved metabolic flux would, however, be required to confirm a shift toward oxidative phosphorylation.

This metabolic shift in hCMEC/D3 grown with 0.25% FBS appeared to occur after three to four weeks of cultivation, when the targeted shear stress of 5.2 dyne/cm^2^ was reached (Figure 2). This level of shear stress was selected because it approximates physiological shear forces present in brain capillaries *in vivo*, positively affecting barrier properties (5–20 dyne/cm^2^) [24,26,28,29]. Similar metabolic transitions have been reported in other dynamic *in vitro* BBB models employing hCMEC/D3, human microvascular endothelial cells (HBMECs), or bovine aortic endothelial cells (BAEC), where a shear stress of around 4 dyne/cm^2^ induced a shift toward aerobic metabolism [35,36,51]. These findings underscore the important role of shear stress in modulating the intracellular bioenergetic pathways of endothelial cells. Interestingly, in our study, this shift occurred only when a low serum concentration was combined with physiological shear stress. hCMEC/D3 exposed to 5.2 dyne/cm^2^ shear stress but cultured in 5% FBS maintained anaerobic respiration throughout the experiment. This suggests that serum reduction is a necessary co-factor for shear stress-induced metabolic reprogramming in this model. In this context, it cannot be excluded that a previously published lactate/glucose ratio of approximately one with hCMEC/D3 cells cultured under flow in a hollow-fiber model with 2% FBS over a time-course of 14 days maybe resulted from still-proliferating cells consuming glucose and the produced lactate due to their increased energy demand [35]. In this regard, observed higher TEER values of hCMEC/D3 cells cultivated with higher FBS amounts might reflect, rather, multilayer formation than barrier differentiation. This would also be in line with our observations, where hCMEC/D3 at 0.25% FBS exhibited in the first cultivation weeks lower TEER values than hCMEC/D3 cells at 1 and 5% FBS, which was reversed for 0.25% FBS conditions later after reaching the physiologically relevant shear stress of 5.2 dyne/cm^2^.

Further on, a former study by Hinkel et al. showed that differences in the gene expression in hCMEC/D3 can occur due to cultivation in a serum-free medium [78]. Therefore, the impact of serum reduction on the gene expression of hCMEC/D3 cultivated in the DIV model was investigated with high-throughput qPCR. Indeed, mRNA level changes dependent on serum reduction were revealed, whereby hCMEC/D3 cultured with 0.25% FBS showed increased mRNA levels of the targets AQP-3, BCRP, CK-18, JAM-2, LRP-8, MCT-8, MRP5, and RXRα and downregulated the mRNA levels of the targets MFSD2A, MRP3, and MUC-18, compared to cells grown with 5% FBS. The targets ABCB1 and ZO-3 were regulated in a more complex manner. These regulated targets have different roles in BBB integrity and brain homeostasis. For instance, JAM-2 and ZO-3 are both components of the tight-junction complex, which seals the paracellular gap between endothelial cells of the BBB [11,79,80,81]. The strong upregulation of JAM-2 and ZO-3 in hCMEC/D3 in 0.25% FBS compared to 5% FBS could indicate an increase in the sealing of the paracellular gap. CK-18 is an intermediate filament of the cytoskeleton that is primarily expressed in single-layered epithelial tissues [82,83]. Thus, the upregulation of CK-18 in hCMEC/D3 cultivated in 0.25% FBS could point to the formation of a mono-layer within the fibers of the DIV model, reducing cellular overgrowth. Moreover, alterations in the gene expressions of several transporter proteins that regulate nutrient influx and efflux (LRP-8, MCT-8, ABCB1, BCRP, MRP3 and MRP5, AQP-3 and MFSD2A) were revealed, indicating a shift towards the reliance on a different set of nutrients supporting the hypothesis of an altered metabolism due to serum reduction [84,85,86,87].

It should be noted that shear stress can also induce transcriptomic changes in TJ and transporter proteins, which, in turn, can lead to altered barrier properties and an increase in maturation [11,26,27,36,88,89,90,91]. For instance, alterations of the mRNA levels of the transporters ABCB1, BCRP, MRP3 and MRP5, as well as the TJ protein ZO-3, occurred in hCMEC/D3 cultured in 0.25% FBS under dynamic conditions. The decrease in gene expression of the cell-surface glycoprotein MUC-18, which is upregulated in the endothelial cells of the BBB during embryonic development [92,93], could further indicate an increase in maturation in hCMEC/D3. Interestingly, changes in the mRNA levels of retinoid X receptors (RXRs) were revealed as well. Retinoic acid, a ligand of RXRs, can increase fatty acid oxidation, which converts fatty acids into acetyl-CoA [94,95,96,97,98]. In hCMEC/D3 cultivated in 0.25% FBS, the gene expression of the subtype RXRα was increased, which could indicate an increase in fatty acid oxidation due to serum reduction. Additionally, RXR is linked to an increase in barrier tightness, as retinoic acid secreted by astrocytes in BBB development causes BBB maturation *in vivo* [7,93]. With regard to transporters, it could be speculated that the upregulation of BCRP and downregulation of ABCB1 might also reflect changes towards a more *in vivo*-like phenotype, since BCRP is more strongly expressed at the human BBB in comparison to ABCB1 [94]. Due to the postulated important role of MFSD2A for the transport of bound fatty acids, such as LPC-DHA, across the BBB [95], it is unclear how this reduction could be interpreted. It has to be mentioned that the serum-reduction possibly did not uniformly enhance all aspects of BBB differentiation or maturity. At the same time, this alteration of MFSD2A expression might be an adaptation to less provided fatty acid concentrations in the growth medium due to FBS reduction. In order to obtain a more comprehensive understanding in this regard also, the expression of other fatty acid transporters such as SLC27A1 should be investigated in the future.

Inflammation is a hallmark of several cerebral diseases, such as stroke and multiple sclerosis, whereby the contribution of the BBB to an inflammatory response in the brain parenchyma is currently highly investigated [1,4,99,100]. Therefore, the second aim of this study was to assess the suitability of hCMEC/D3 cultured under optimized conditions, with 0.25% FBS in the DIV hollow fiber model as a disease model for systemic inflammatory responses in BCEC. Systemic inflammation and circulating cytokines such as TNF-α, IL-1β and IFN-γ can activate endothelial cells of the BBB, inducing an inflammatory response, which is hypothesized to reduce BBB integrity and cause BBB breakdown [1,4,5]. Whereas, in healthy humans, none to very low levels of the circulating cytokines are detected, a 10 to 20 times increase in the circulating cytokines TNF-α IL-1β, and IFN-γ (between 3 and 800 pg/mL) is measured in patients suffering from sepsis or rheumatoid arthritis [101,102]. Interestingly, several *in vitro* models studying the effects of cytokines on the BBB rely on short-term set-ups with an exposure to non-physiological high concentrations of proinflammatory cytokines such as TNF-α, IL-1β, IFN-γ and IL-6 (e.g., 10 ng/mL and higher) for 24–72 h [103,104,105,106,107,108,109,110,111,112,113,114]. Although some *in vitro* BBB models were used to investigate the effects at lower cytokine concentrations, to our best knowledge, almost none of these studies had been conducted for longer periods [56,57,58,59]. Therefore, for the development of a more organotypic DIV model, it was investigated whether a significant inflammatory response could be induced at lower, more physiologically relevant cytokine concentrations and whether this inflammatory response could be prolonged with dynamic culture conditions. Previous studies with hCMEC/D3 or primary human brain microvascular endothelial cells cultivated under dynamic conditions demonstrated that treatment with 1 ng/mL up to 100 ng/mL TNF-α for two hours up to 24 h led to a decrease in TEER, an increase in the permeability of paracellular markers or in the expression of proinflammatory cytokines such as IL-6 in endothelial cells [88,105,115,116,117]. In this study, a decrease in TEER was only observed for the higher CK concentration of 10 ng/mL after treatment for two weeks, but no changes in FD4 permeability at any time point or CK concentration were determined. In light of the discrepant findings across these different studies, the discussion about the relevant and appropriate CK concentration for mimicking systemic inflammatory responses in *in vitro* BBB experiments should also consider aspects about the extent of BBB breakdown observed *in vivo* during chronic/longer-term systemic inflammation and the complexity of cytokine mixtures.

As a response to circulating cytokines, activated endothelial cells express proinflammatory cytokines and chemokines such as IL-6, IL-8, and MCP-1, as well as TNF-α and IL-1β, creating an inflammatory environment that further propagates inflammation to surrounding cells, including astrocytes and pericytes. Astrocytes and pericytes may also enter a reactive state upon exposure to TNF-α, IL-1β, and IFN-γ, as well as to IL-6, IL-8, and MCP-1. Depending on the proinflammatory cue, astrocytes and pericytes themselves can secrete TNF-α, IL-1β, IL-6, IL-8 and MCP-1, contributing to the inflammatory environment at the BBB and the brain parenchyma [105,118,119,120,121,122,123,124].

In this study, a short-term experiment for 24 h with Transwells^®^ revealed that, upon apical CK treatment, the apically seeded hCMEC/D3, as well as the basolateral hAP, showed an increase in the secretion of IL-6 and MCP-1 after 24 h of CK treatment in regard to CK concentration. Interestingly, IL-8 was only significantly increased in apical samples, which could indicate that hCMEC/D3 only secrete IL-8 toward the “blood side”. Moreover, IL-6 was only significantly increased in basolateral samples of hCMEC/D3 in triple-culture with hAP and not in basolateral samples of mono-cultures. This could indicate that astrocytes or pericytes also secrete IL-6 in response to factors secreted or transported by hCMEC/D3 from the apical side to the basolateral side. Due to the differing results obtained for mono-cultures compared to triple-cultures, the necessity of the triple-culture set-up for the DIV-model experiments was demonstrated. After proving that hCMEC/D3, as well as hAP, cultivated in static conditions respond to the inflammatory stimuli by secreting IL-6, IL-8 and MCP-1, it was investigated as to whether these inflammatory responses could be sustained under dynamic conditions for up to two weeks. Indeed, hCMEC/D3 cultivated in the DIV model as a triple-culture with hAP secreted IL-6, IL-8 and MCP-1 as a response to the exposure to TNF-α, IL-1β and INF-γ as well, as indicated by a significant increase in cytokine and chemokine concentrations in samples collected from the medium reservoir and the ECS. As in Transwells^®^, the secretion levels of IL-6, IL-8 and MCP-1 correlated with the CK concentration. The secretion levels of IL-6, IL-8 and MCP-1 were quite similar at the “blood side” and ECS throughout the experiment, which could indicate that hAP entered a reactive state in response to the proinflammatory factors contributing to the increased levels of IL-6, IL-8 and MCP-1 in the ECS.

To investigate the underlying molecular changes on the gene expression level of BBB markers of hCMEC/D3 during inflammatory response, high-throughput qPCR was performed. In the Transwell^®^ model, 24 h CK treatment of hCMEC/D3 cultivated as a triple-culture with hAP revealed the upregulation of the mRNA levels of the surface adhesion molecules VCAM-1 and ICAM-1, a well-known response of brain endothelial cells to inflammation [102,125], as well as the upregulation of JAK-1, which is a downstream protein of the TNF-α/NFκB signaling pathway [60]. Moreover, CLDN-5, ZO-1, and occludin, which are highly abundant proteins contributing to TJ complexes at the BBB, were significantly regulated in hCMEC/D3 cultivated in Transwells^®^, emphasizing the influence of proinflammatory cytokines on barrier integrity. It should be mentioned that significant differences in the gene expression of several targets between mono-cultures and triple cultures with hAP were observed, indicating that microenvironmental cells can contribute to the inflammatory state of endothelial cells. The analysis of changes in the gene expression of hCMEC/D3 cultivated in the DIV model exposed to CKs revealed a complex, dynamic regulatory network. Compared to controls, the targets AQP-5, BCRP, CLDN-6, CLDN-18, CLDN-22 and vWF were downregulated after CK treatment, independent of CK concentration, whereas CLDN-5 was regulated dependent on the CK concentration. A reduced expression of the selective water channel AQP-5 was recently described in epithelial cells during pulmonary inflammation and after acute viral infection, indicating that AQP-5 expression might be regulated during cellular proinflammatory responses [126,127]. BCRP is an efflux transporter highly conserved at the human BBB, which was shown to be downregulated upon exposure to IL-1β in porcine and human *in vitro* BBB models, which is consistent with the data of the current study [128]. CLDN-18, a TJ protein expressed in the kidney, the lung and at the BBB, was shown to be downregulated when IL-1β expression was upregulated in an *in vitro* lung model [41,129,130]. These results might indicate that, in our models, IL-1β contributed to the proinflammatory response. CLDN-6 and CLDN-22 are both expressed in brain endothelial cells, and CLDN-6 acts as a TJ protein for paracellular sealing [41,131,132]. The role of CLDN-22 is not yet fully understood, but it is a homologue to CLDN-18, which could indicate that it also might be able to function as a sealing TJ protein [129,131]. Interestingly, CLDN-5, one of the most prominent TJ proteins at the BBB, was significantly upregulated in hCMEC/D3 after 0.1 ng/mL CK treatment but significantly downregulated in hCMEC/D3 after 10 ng/mL CK treatment in both the Transwell^®^ and DIV model. The downregulation of CLDN-5, CLDN-6, CLDN-18 and CLDN-22 due to CK treatment could suggest that these TJ proteins might contribute to BBB breakdown during systemic inflammation.

Comparison of the short-term Transwell^®^ model with the longer-term DIV model revealed both concordant and divergent regulations in gene expression. Several targets were regulated similarly in hCMEC/D3 across both platforms, including AQP-5, BCRP, CLDN-5, CLDN-10, CLDN-12, occludin and ZO-1. However, some differences were also observed. For example, CLDN-23 and MCT-1were upregulated in the Transwell^®^ model but were not significantly regulated in the DIV model. Likewise, the inflammatory markers ICAM-1, ICAM-2, and VCAM-1 were significantly induced in the Transwell^®^ cultures but did not show significant regulation in the DIV system. It should be noted that the lower statistical power in the DIV model, resulting from the smaller sample size, may have limited the detection of more subtle regulatory effects. Some differences in mRNA levels might be a result of the longer CK treatment time (24 h vs. two weeks) in the DIV model and an adaptation of the cells over time. Interestingly, no differences in the expression of inflammation markers due to shear stress were revealed when comparing control triple-cultures in the Transwell^®^ system to the DIV system (Table S19).

In general, when comparing the Flocel system to the Transwell^®^ models, it has to be mentioned that, due to its complexity and high maintenance demands, the Flocel model is limited in the number of systems that can be operated in parallel, rendering it unsuitable for high-throughput applications. Thus, static Transwell^®^ systems are more appropriate for large-scale studies, such as compound screening, due to their higher parallelizability and easy maintenance. But, in contrast to DIV models, static Transwells^®^ are constrained by limited culture duration, as blood–brain barrier integrity is not as stable after a few days [51,78]. By the continuous flow of medium along the endothelial cells, the Flocel system not only allows the optimum nutrient supply (and waste disposal) by constant medium delivery but also resembles *in vivo* conditions by inducing shear stress, which benefits the barrier establishment and increases barrier integrity and, in turn, enables a culture duration for up to weeks to months. Thus, the Flocel system is more suitable for long-term studies of pathophysiological conditions such as inflammation [25,31,133,134,135,136].

Moreover, although the Flocel system, as a millifluidic platform, enables the generation of substantially larger sample quantities than microfluidic systems, facilitating robust gene expression and metabolomic analyses, the amount of recovered material remains restricted for certain comprehensive protein-based assays needing high protein amounts, including Western blotting. In addition, immunofluorescence-based characterization is complicated due to the non-transparent nature and the background fluorescence of the fibers and the limited accessibility of the hollow fibers embedded within the Flocel cartridge. Their manipulation is technically challenging and carries a high risk of disrupting the intact cellular layer, thereby compromising sample integrity. Current developments in next-generation millifluidic platforms aim to address limitations in optical accessibility and compatibility with fluorescence-based imaging approaches [137]. In this regard, a recent publication introduced transparent polymeric hollow fibers, confirming short-term monolayer cultivation of hCMEC/D3 cells [138]. However, despite these limitations, functional assessments provide the most physiologically relevant readouts, while targeted gene expression profiling offers complementary molecular information that supports the interpretation of the observed functional responses.

Accurate *in vitro* modeling of systemic inflammation also requires the inclusion of immune cells, which represent a critical component of the inflammatory response [55,135]. During systemic inflammation, various immune cells, such as leucocytes, natural killer cells, neutrophils, and monocytes, may be recruited by activated BCEC to cross the BBB and eventually enter the brain parenchyma, further propagating the inflammatory cascade and contributing to tissue damage [139,140,141,142,143]. Thus, this study might be limited in fully recapitulating inflammatory cues, which would have been propagated or demolished by pro- and anti-inflammatory cytokines and chemokines secreted by infiltrating immune cells.

In summary, although limited by the absence of immune cells, the established BBB DIV model offers several advantages. The applied flow allows for long-term cultivation under shear stress and optimized culture conditions, which permits the investigation of system inflammation at physiologically relevant cytokine levels over periods of several weeks. In addition, as the DIV model comprises two distinct compartments that are connected, cytokine exposure and downstream inflammatory responses can be assessed on both the vascular (“blood”) and parenchymal (“brain”) sides, and bidirectional signaling and interactions between the different cell types can be assessed.

## 5. Conclusions

The major outcome of this study is that a physiologically more relevant DIV model of the BBB for studying systemic inflammation can be established by optimizing culture conditions for the human hCMEC/D3 cell line. Serum reduction from 5% FBS to 0.25% FBS led to a decrease in proliferation of hCMEC/D3 without compromising barrier integrity. Furthermore, cultivation in 0.25% FBS induced a metabolic shift from predominantly anaerobic glycolysis to presumably aerobic respiration. This shift was supported by significant changes in the consumption and secretion of key metabolites involved in energy metabolism and the TCA cycle, suggesting a greater reliance on mitochondrial ATP production (Figure S10: TCA cycle scheme with an overview of where the measured metabolites interact). Moreover, regulation of the gene expression of BBB-relevant targets, such as TJ and transporter proteins, indicated that serum reduction also affected BBB markers in hCMEC/D3 at the molecular level. Another key finding was that hCMEC/D3 co-cultured with hAP under dynamic conditions in 0.25% FBS were susceptible to physiologically relevant low cytokine concentrations of 0.1 ng/mL TNF-α, IL-1β and IFN-γ, which pushed hCMEC/D3 into an active state. While barrier breakdown was observed only at the higher cytokine concentration (10 ng/mL CK), the lower concentration (0.1 ng/mL CK) was sufficient to trigger endothelial proinflammatory responses and the secretion of significant amounts of the proinflammatory cytokines IL-6, IL-8, and MCP-1. Changes in mRNA levels due to CK treatment with 0.1 ng/mL and 10 ng/mL revealed a complex regulatory network of intracellular processes triggered by the exposure to cytokine stimuli. Comparison of these data with the results obtained from the static Transwell^®^ model further highlighted the potential of the DIV Flocel BBB model to mimic systemic inflammation over an extended period, utilizing the effects of shear stress, and thereby enabling long-term experiments under more physiologically relevant conditions. Future studies might include more validation at the protein and transporter functional level, but also integrate larger metabolite panels, isotope tracing, OCR/ECAR measurements, and network-based multi-omics analyses to confirm the proposed mechanisms.

## Data Availability

The data presented in this study are available on request from the corresponding author.

## Supplementary Materials

The following supporting information can be downloaded at https://www.mdpi.com/article/10.3390/pharmaceutics18070817/s1: Supplementary Figures (S1–S10) and Tables (S1–S19). Figure S1: The increase in shear stress over time on the cell layer of hCMEC/D3 cultured within the fibers of the DIV cartridge is indicated in this graph; Figure S2: Serum-dependent cell numbers and RNA concentration of hCMEC/D3 cultivated as mono-culture in the DIV model; Table S1: Description of the preparation of the cytokine stocks of TNF-α, IL-1β and INFγ in 0.1% BSA; Table S2: Average of counted cell number per week after weekly medium change of Flocel mono-cultures per culture condition with different serum concentrations of 5% FBS, 1% FBS or 0.25% FBS; Table S3: Average of total cell number collected after weekly medium change of DIV-model mono-cultures per culture condition with different serum concentrations of 5% FBS, 1% FBS or 0.25% FBS; Table S4: RNA amount of harvested hCMEC/D3 from the fibers of the DIV cartridges at end of experiment with differing serum concentrations of 5% FBS, 1% FBS and 0.25% FBS; Table S5: Average values of glucose consumption [mmol/day] and lactate production [mmol/day] of hCMEC/D3 in DIV-model mono-culture cultivated in different serum concentrations of 5% FBS or 0.25% FBS per week; Figure S3: Changes in metabolite levels after cultivation for one week, four weeks and seven weeks. Table S6: Variations in extracellular metabolites at week 1 expressed as fold change versus acellular medium. Data are presented as average fold change (FC) ± SEM (FC); Table S7: Variations in extracellular metabolites at week 4 expressed as fold change versus acellular medium; Table S8: Variations in extracellular metabolites at the final time point expressed as fold change versus acellular medium; Table S9: Average of TEER measurements of DIV-model mono-culture hCMEC/D3 cultured in 5% FBS, 1% FBS, or 0.25% FBS; Table S10: Permeability coefficient (PC) of the paracellular marker FD4 from the fibers to the ECS of the DIV-model mono-culture with hCMEC/D3 cultivated in 5% FBS compared to 1% FBS and 0.25% FBS; Figure S4: Heat map with hierarchical clustering of targets (log2(FC)) measured with high-throughput qPCR for samples of hCMEC/D3 cultured in the DIV model as mono-cultures with 5%, 1% or 0.25% FBS; Table S11: x-fold values of gene expression changes detected with high throughput qPCR of Flocel mono-cultures cultivated in different serum concentrations of 5%, 1% or 0.25%FBS at the end of the cultivation period; Table S12: Average TEER of hCMEC/D3 cultivated in the DIV model as triple-culture with human primary astrocytes and pericytes (hAP); Table S13: permeability coefficient of paracellular marker FD4 for permeability studies of hCMEC/D3 cultivated in the DIV model as triple-culture with human primary astrocytes and pericytes; Figure S5: Proof of principle experiment with Transwells^®^. To determine if hCMEC/D3 are responsive to the CK treatment with 0.1 ng/mL or 10 ng/mL TNF-α, IL-1β and INFγ, hCMEC/D3 were cultivated on Transwells^®^; Table S14: Concentration of proinflammatory cytokines IL-6, IL-8 and MCP-1 in Transwells^®^ cultured with hCMEC/D3 in mono-culture (mono) or in triple-culture with human primary astrocytes and pericytes (hAP) at different time points as a response to treatment with the cytokine cocktail of TNF-α, IL-1β and INF-γ with a concentration of 0.1 ng/mL or 10 ng/mL each or to control treatment with 0.1% BSA; Table S15: Concentration of proinflammatory cytokines IL-6, IL-8 and MCP-1 in fibers of the DIV-model triple culture with hCMEC/D3, human primary astrocytes and pericytes at different time points as a response to treatment with the cytokine cocktail of TNF-α, IL-1β and INF-γ with a concentration of 0.1 ng/mL or 10 ng/mL each or to control treatment with 0.1%BSA; Table S16: Concentration of proinflammatory cytokines IL-6, IL-8 and MCP-1 in the ECS of the DIV-model set-ups cultured with hCMEC/D3, human primary astrocytes and pericytes at different time points as a response to treatment with the cytokine cocktail of TNF-α, IL-1β and INF-γ with a concentration of 0.1 ng/mL or 10 ng/mL each or to control treatment with 0.1%BSA; Figure S6: The secretion of proinflammatory cytokines IL-6, IL-8 and MCP-1 by human astrocytes and pericytes (hAP) cultivated in the ECS surrounding the fibers of the DIV model was determined; Figure S7: Heat map with hierarchical clustering of targets (log2(FC)) measured with high-throughput qPCR for samples of hCMEC/D3 cultured in Transwells^®^ as mono-culture (mono) or triple culture with human primary astrocytes and pericytes (hAP); Figure S8: Heat map with hierarchical clustering of targets (log2(FC)) measured with high-throughput qPCR for samples of hCMEC/D3 cultured in Transwells^®^ as triple culture with human primary astrocytes and pericytes (hAP); Table S17: x-fold values of high throughput qPCR of hCMEC/D3 cultivated on Transwells^®^ in mono-culture or in co-culture with human astrocytes and pericytes (hAP) for 24 h with 0.1 ng/mL or 10 ng/mL CK cocktail consisting of TNF-a, IL-1b or IFN-g each; Figure S9: Heat map with hierarchical clustering of targets (log2(FC)) measured with high-throughput qPCR for samples of hCMEC/D3 cultured in the DIV model as triple-cultures after 14 days treatment with 0.1 ng/mL or 10 ng/mL TNFα, IL-1β and INFγ, or 0.1% BSA as control; Table S18: x-fold values of high throughput qPCR of hCMEC/D3 cultured in the DIV model as triple-cultures cultivated with a serum concentration of 0.25%FBS; Table S19: Comparison of the gene expression of inflammatory markers in hCMEC/D3 in control triple-cultures of the Transwell^®^ system to the Flocel system. Figure S10: Schematic drawing of the TCA cycle and relevant metabolites that were regulated during serum dependency experiments and inflammation experiments.

## Abbreviations

The following abbreviations are used in this manuscript:

3-HIB	3-hydroxyisobutyrate
ABC	ATP-binding cassette
AJ protein	adherens junction protein
AM	astrocyte Medium
ATP	adenosintriphosphate
BAEC	bovine aortic endothelial cells
BBB	blood–brain barrier
BCAA	branched-chain amino acids
BCAT	branched-chain aminotransferases
BCEC	brain capillary endothelial cells
BCKAs	branched-chain α-keto acids
BCRP	breast cancer resistance protein
BPM	beats per minute
BSA	bovine serum albumin
CK	cytokine
Coll-IV	collagen IV
DIV model	dynamic in vitro model
EAAT	excitatory Amino Acid Transporter
EBM-2	endothelial basal medium-2
ECAR	extracellular acidification rate
ECS	extracapillary space
FBS	fetal bovine serum
FC	fold change
FD4	fluorescein isothiocyanate-dextran, 4 kDa
Fibro/Coll-IV	fibronectin/collagen-IV
FITC	fluorescein isothiocyanate
hAP	human primary astrocytes and pericytes
HBMEC	human microvascular endothelial cells
hCMEC/D3	human brain microvascular endothelial cell line
KIC	α-ketoisocaproate
KIV	α-ketoisovalerate
KMV	α-keto-β-methylvalerate
LSR	angulin-1
MC	medium change
MMP	metalloproteinase
MRP	multidrug-resistance related proteins
NMR spectroscopy	nuclear magnetic resonance spectroscopy
OCR	oxygen consumption rate
PBMEC/C1-2	porcine brain microvascular endothelial cell line
PBS	phosphate-buffered saline
Pe-values	effective permeability values
PgP	P-glycoprotein
P-L-L	poly-L-lysine
PM	pericyte medium
RxR	retinoic-X-receptor
SLC	solute carrier
TCA cycle	tricarboxylic Acid (TCA) cycle
TEER	transendothelial electrical resistance
TJ protein	tight-junction protein
ZO-1	zonula occludens 1

## Appendix A

### Appendix A.1

70% EtOH activation and washing steps of the DIV-BBB cartridge:

For activating the hollow fibers of the DIV-BBB cartridge, sterile syringes (3 mL syringe for port 2 and 3, 10 mL syringes for port 1 and 4, Becton Dickinson, Franklin Lakes, NJ, USA, Ref.: 309658 or 300912) filled with either 3 mL or 7 mL of 70% sterile EtOH were mounted to port 2 or port 1, whereas an empty sterile 10 mL syringe and an empty sterile 3 mL syringe was mounted to port 4 or port 3 (Figure A1B). First, the lumen was filled with EtOH by slowly pushing downwards on the plunger of the filled syringe mounted to port 1 and simultaneously pulling upwards the plunger of the empty syringe mounted on port 4 in a constant movement. Then, the ECS was filled with 1 mL of 70% EtOH as well by slowly pushing downwards the plunger of the filled syringe mounted to port 2 and simultaneously pulling upwards the plunger of the empty syringe mounted on port 3 in a constant movement. When both, lumen and ECS, were completely filled with 70% sterile EtOH, the liquid was moved within the lumen and the ECS by simultaneous pushing and pulling the plungers of ports 1 and 4 for five to seven times, then by moving the plungers of ports 2 and 3 for five to seven times, and finally by moving the plungers of the syringes mounted to ports 1 and 3 and ports 2 and 4 to ensure liquid transfer through the wholes of the hollow fibers from the lumen to the ECS and vice versa. Fibers were incubated in 70% EtOH for at least 2 min. After fiber activation, the EtOH was removed completely from the lumen and the ECS, by first turning the cartridge upside down and then by collecting the EtOH in the syringe mounted to port 1 and port 2 by pushing the plunger of the syringe mounted to port 4 or port 3 to 0 mL and simultaneously pulling the plungers of the syringes mounted to ports 1 and 2. Then, the cartridge was turned back, and the EtOH-filled syringes on ports 1 and 2 were replaced, respectively, with an air-filled 10 mL and 3 mL syringe. Again, to collect the residual EtOH, the cartridge was turned upside down. By pushing down the plunger of the completely air-filled syringe mounted to port 1 and simultaneously pulling the plunger of the syringe mounted to port 4, the residual EtOH in the lumen is collected, followed by pushing and pulling of the plungers of the syringes mounted to ports 2 and 3 to remove the remaining EtOH from the ECS. To remove residual EtOH from the system, the lumen and the ECS were rinsed two times with 10 mL sterile Millipore-H2O by plunger pulling and pushing as described above for the EtOH activation. Afterwards, the fibers were coated with 5 mL of a mixture of Collagen IV (0.1 mg/mL) and Fibronectin (1.0 mg/mL) in sterile PBS or H2O overnight at 37 °C.

### Appendix A.2

Seeding hCMEC/D3 into the fibers of the DIV-BBB cartridge:

In detail, the syringe containing the cell suspension (1.5 × 10^6^ hCMEC/D3 in 5 mL) was mounted onto port 1, whereas an empty 5 mL syringe was mounted onto port 4. The cell suspension was moved along the fibers five to seven times in a slow and steady movement by pushing and pulling the plungers of the syringes mounted to the luminal ports 1 and 4. Then, the cell suspension was moved towards the ECS by alternating pulling the plungers of the empty syringes mounted to the ECS ports 2 and 3 and by alternating pushing of the plungers of the syringes mounted to the luminal ports 1 and 4 to ensure that all cells are located within the fibers. As the endothelial cells are restricted from entering the ECS by the small pore sizes of the fibers, only medium enters the ECS. After the seeding procedure, the syringes were removed, and the ports were sealed with plugs (B. Braun, Austria, Ref.: BD394080).
