# Supplementary material for: Establishment and Optimization of a Human Flow-Based Hollow Fiber In Vitro Blood–Brain Barrier Model for Systemic Inflammatory Responses
Source: Pharmaceutics. 2026 Jun 30;18(7):817. doi: 10.3390/pharmaceutics18070817 (PMC13414653; doi:10.3390/pharmaceutics18070817)
Supplement: Supplementary file 1 [file pharmaceutics-18-00817-s001.zip › pharmaceutics-4311997-supplementary (2).pdf]

Supplementary part

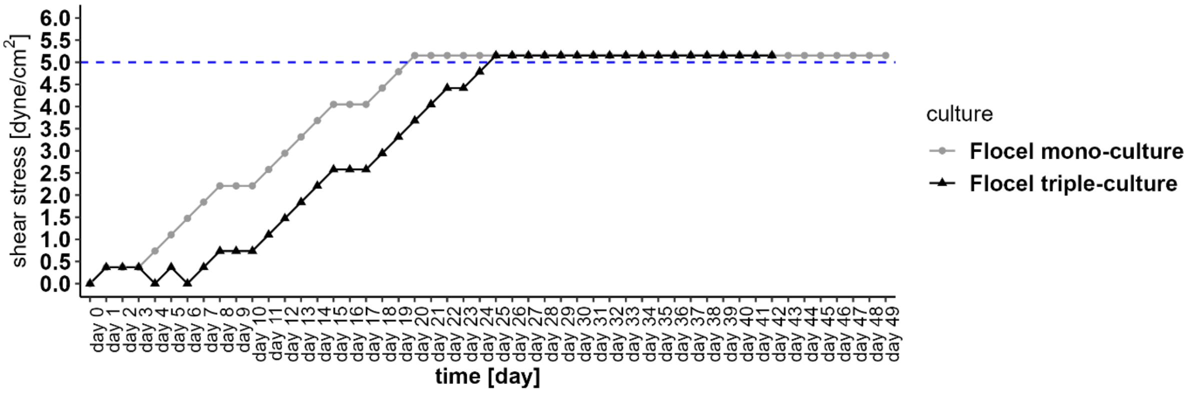

Figure S1: The increase in shear stress over time on the cell layer of hCMEC/D3 cultured within the fibres of the DIV-cartridge is indicated in this graph. The constant increase in flow rate was proportional to the increase in shear stress. A shear stress of 5 dyne/cm^2 is believed to recapitulates in-vivo conditions at the BBB and should induce BBB-characteristics. For DIV-model mono-cultures a shear stress of 5.2 dyne/cm^2 was reached at day 20, whereas for DIV-model triple-cultures a shear stress of 5.2 dyne/cm^2 was reached after 26 days in cultivation. The blue line is set at 5 dyne/cm^2.

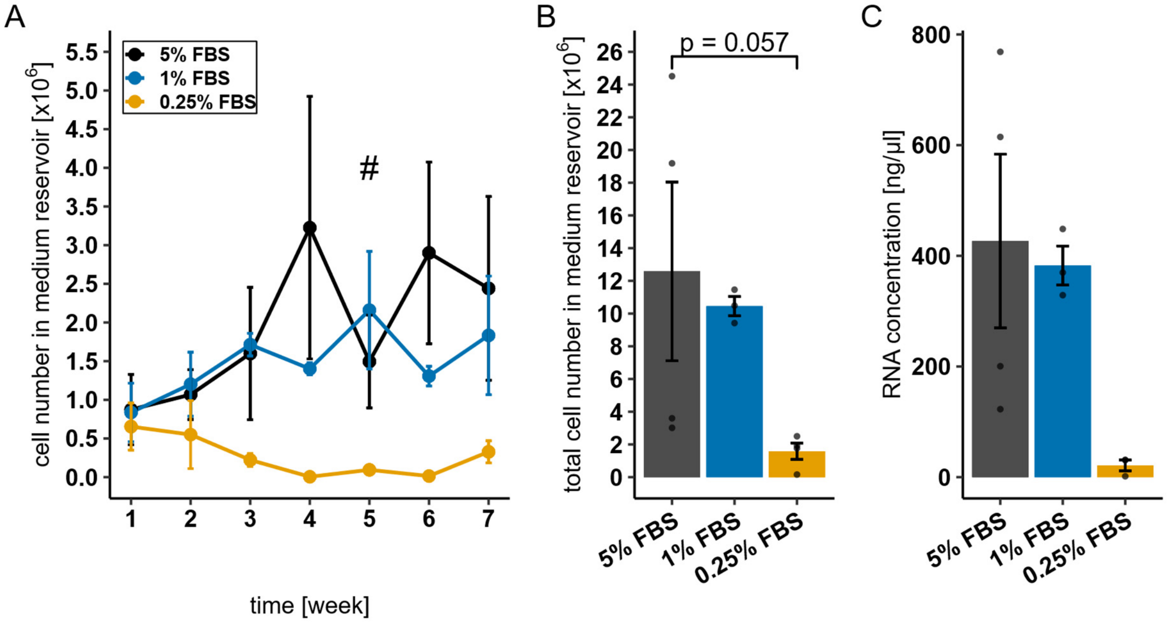

Figure S2: Serum-dependent cell numbers and RNA concentration of hCMEC/D3 cultivated as mono-culture in the DIV-model. (A) The number of cells collected from the medium reservoir of hCMEC/D3 mono-cultures in the DIV-model cultivated with 5% FBS, 1% FBS or 0.25% FBS was counted after the weekly medium change. A reduced number of cells was determined for cultures with 0.25% FBS (n=5-7, N=2-4). (B) The assessment of the total number of cells collected from the medium reservoir after the weekly medium change from week 1 to week 7 revealed an overall reduced cell number of hCMEC/D3 mono-cultures cultivated with 0.25% FBS (n=5-7, N=2-4). (C) The RNA concentration of hCMEC/D3 harvested at the experiment end point showed a reduction in RNA amount dependent on the serum concentration (n=3-4, N= 3-4). Data are presented as mean ± SEM. One-way ANOVA with Dunn's Method for post-hoc testing was applied. $p<0.05$; # indicates significances against 1% FBS.

Table S1: Description of the preparation of the cytokine stocks of TNF-α, IL-1β and INFγ in 0.1% BSA. The first dilution step was prepared according to manufaturer's instructions, when available. After the final concentration of 1 µg/ml was reached, the stock solutions were sterile-filtered with a rotilabo filter (0.22 µm, PVDF, Carl Roth, Germany, Ref.: P666.1).

Cytokine	First dilution step			Stock solution	
	Powder	Diluted in	Conc.	Dilution step	Final conc.
TNF-α	10 µg	1000 µl 0.1% BSA	10 µg/ml	1 ml of 10 µg/ml<br>+ 9 ml of 0.1% BSA	1 µg/ml
IL-1β	2 µg	20 µl 0.1% BSA	100 µg/ml	20 µl of 100 µg/ml<br>+ 1.98 ml 0.1% BSA	1 µg/ml
INF-γ	20 µg	200 µl 0.1% BSA	100 µg/ml	200 µl of 100 µg/ml<br>+ 19.8 ml 0.1% BSA	1 µg/ml

Table S2: Average of counted cell number per week after weekly medium change of Flocel mono-cultures per culture condition with different serum concentrations of 5% FBS, 1% FBS or 0.25% FBS (n=4-7, N=3-4). Data are presented as mean ± SEM. One-Way ANOVA with Dunn's Method for post-hoc testing, p<0.05, # significant against 1% FBS.

Time [week]	Flocel mono-culture 5% FBS (*10^6 cells)	Flocel mono-culture 1% FBS (*10^6 cells)	Flocel mono-culture 0.25% FBS (*10^6 cells)
Week 1	0.87 ± 0.37	0.84 ± 0.31	0.77 ± 0.33
Week 2	1.07 ± 0.28	1.20 ± 0.34	0.55 ± 0.31
Week 3	1.60 ± 0.74	1.71 ± 0.12	0.22 ± 0.07
Week 4	3.23 ± 1.39	1.40 ± 0.07	0.01 ± 0.004
Week 5	1.49 ± 0.52	2.16 ± 0.62	0.09 ± 0.04 **#**
Week 6	2.90 ± 1.02	1.31 ± 0.10	0.01 ± 0.01
Week 7	2.44 ± 1.03	1.83 ± 0.63	0.33 ± 0.12

Table S3: Average of total cell number collected after weekly medium change of DIV-model mono-cultures per culture condition with different serum concentrations of 5% FBS, 1% FBS or 0.25% FBS (n=4-7, N=3-4). Data are presented as mean ± SEM. One-Way ANOVA with Dunn's Method for post-hoc testing, p<0.05, * significant against 5% FBS, # significant against 1% FBS.

Flocel [no.]	Flocel mono-culture 5% FBS (*10^6 cells)	Flocel mono-culture 1% FBS (*10^6 cells)	Flocel mono-culture 0.25% FBS (*10^6 cells)
#1	3.02	11.46	1.91
#2	24.51	10.49	2.50
# 3	19.19	9.42	1.76
# 4	3.60	n.a.	0.16
average	12.58 ± 4.73	10.46 ± 0.48	1.58 ± 0.43***(p=0.057)**

Table S4: RNA amount of harvested hCMEC/D3 from the fibres of the DIV-cartridges at end of experiment with differing serum concentrations of 5% FBS, 1% FBS and 0.25% FBS. (n=3-4, N=3-4). Mean ± SEM. One-way ANOVA with Dunn's Method for post-hoc testing. p<0.05, * significant against 5% FBS; # significant 1% FBS.

Experiment #	Flocel – 5% FBS [ng/µl]	Flocel – 1% FBS [ng/µl]	Flocel – 0.25% FBS [ng/µl]
#1	200.5	328.9	30.9
#2	768.6	448.5	30.1
#3	614.8	369.8	1.6
#4	122.8	/	/
**Average**	**426.7 ± 273.2**	**382.4 ± 28.7**	**21.2 ± 8.0**

Table S5: Average values of glucose consumption [mmol/day] and lactate production [mmol/day] of hCMEC/D3 in DIV-model mono-culture cultivated in different serum concentrations of 5% FBS or 0.25 % FBS per week. (n=2-4; N=3-4). Mean ± SEM; One-way ANOVA with Dunn's Method for post-hoc testing. $p < 0.05$, * significant against 5% FBS.

Serum concentration	Time	Average Glucose [mmol/day]	Average Lactate [mmol/day]	Lactate / Glucose ratio
5% FBS	week 0	0.00 ± 0.00	0.00 ± 0.00	/
5% FBS	week 1	0.04 ± 0.01	0.05 ± 0.02	1.21
5% FBS	week 2	0.03 ± 0.01	0.09 ± 0.02	3.28
5% FBS	week 3	0.03 ± 0.01	0.10 ± 0.02	3.11
5% FBS	week 4	0.03 ± 0.005	0.10 ± 0.03	3.82
5% FBS	week 5	0.05 ± 0.004	0.11 ± 0.01	2.45
5% FBS	week 6	0.08 ± 0.02	0.12 ± 0.02	1.58
5% FBS	week 7	0.05 ± 0.01	0.12 ± 0.01	2.30
**5% FBS**	**Average (week 1-week 7)**	**0.04 ± 0.01**	**0.10 ± 0.01**	**2.53 ± 0.33**
0.25% FBS	week 0	0.00 ± 0.00	0.00 ± 0.00	/
0.25% FBS	week 1	0.03 ± 0.001	0.03 ± 0.01	1.39
0.25% FBS	week 2	0.01 ± 0.004	0.03 ± 0.01	2.15
0.25% FBS	week 3	0.01 ± 0.004	0.01 ± 0.01 *	1.09
0.25% FBS	week 4	0.02 ± 0.002	0.002 ± 0.002 *	0.11
0.25% FBS	week 5	0.02 ± 0.00 *	0.003 ± 0.003 *	0.16
0.25% FBS	week 6	0.01 ± 0.01	0.000 ± 0.00	0.00
0.25% FBS	week 7	0.01 ± 0.002 *	0.004 ± 0.004 *	0.45
**0.25% FBS**	**Average (week 1-week 7)**	**0.01 ± 0.004 ***	**0.02 ± 0.003 ***	**0.75 ± 0.28 ***

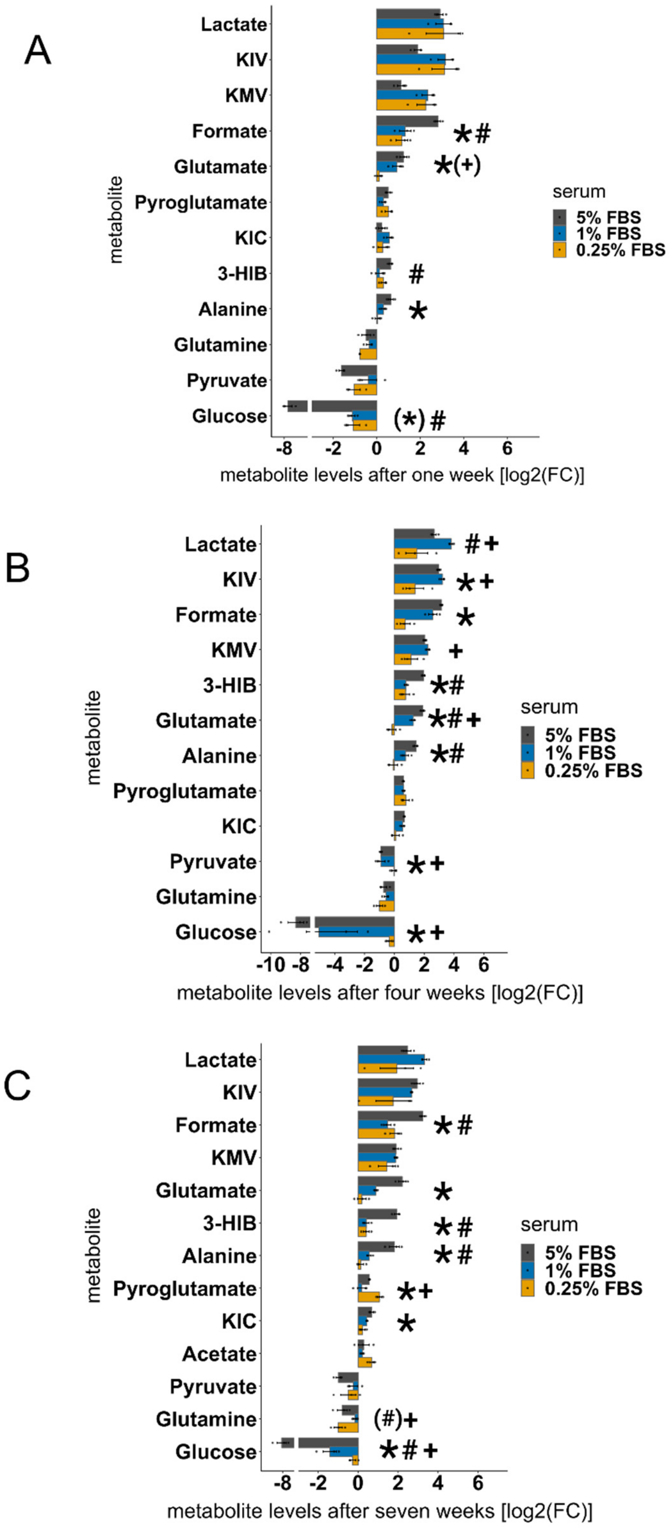

Figure S3: Changes in metabolite levels after cultivation for one week, four weeks and seven weeks. Significant changes in the secretion or consumption of metabolites were detected when comparing samples collected from the medium reservoir during medium change after cultivation of hCMEC/D3 for one week (A), four weeks (B) or seven weeks (C) in 5% FBS, 1% FBS and 0.25% FBS to metabolite levels of acellular medium. Metabolites with a value above 0 are assumed to be secreted, whereas metabolites with a value below 0 are assumed to be consumed (n=2-3, N=2-3). Data are presented as mean ± SEM. One-way-ANOVA with Tukey's HSD for post-hoc testing; $p<0.05$, * 5% FBS significant against 0.25% FBS, # 5% FBS significant against 1%; + 1% FBS significant against 0.25% FBS; () indicates a trending up- or downregulation with $p<0.075$.

Table S6: Variations in extracellular metabolites at week 1 expressed as fold change versus acellular medium. Data are presented as average fold change (FC) ± SEM (FC). Significant differences (P < 0.05) assessed through one-way-ANOVA with Tukey's HSD for post-hoc testing. n.s. indicates metabolites with no significant changes. Medium was changed every week. (N=3; n=3)

metabolite	fibre-w1-5%	fibre-w1-1%	fibre-w1-0.25%	Significant differences
**Glucose**	0.004 ± 0.001	0.463 ± 0.042	0.493 ± 0.110	5% vs 1% (P = 0,008)<br>5% vs 0.25% (P = 0,06)
**Pyruvate**	0.326 ± 0.025	0.826 ± 0.238	0.507 ± 0.104	n.s.
**Glutamine**	0.728 ± 0.101	0.787 ± 0.063	0.583 ± 0.002	n.s.
**α-Ketoisocaproate (KIC)**	1.182 ± 0.116	1.496 ± 0.123	1.243 ± 0.176	n.s.
**Pyroglutamate**	1.438 ± 0.086	1.239 ± 0.070	1.459 ± 0.145	n.s.
**3-Hydroxyisobutyrate (3-HIB)**	1.557 ± 0.068	1.111 ± 0.135	1.245 ± 0.085	5% vs 1% (P = 0,045)
**Alanine**	1.572 ± 0.119	1.236 ± 0.078	1.031 ± 0.083	5% vs 0.25% (P = 0,017)
**α-Keto-β-methylvalerate (KMV)**	2.194 ± 0.241	5.210 ± 0.838	5.121 ± 1.225	n.s.
**Glutamate**	2.355 ± 0.254	1.939 ± 0.255	1.086 ± 0.070	5% vs 0.25% (P = 0,013)<br>1% vs 0.25% (P = 0,066)
**α-Ketoisovalerate (KIV)**	3.696 ± 0.374	9.320 ± 1.870	9.960 ± 3.072	n.s.
**Formate**	7.083 ± 0.568	2.550 ± 0.432	2.302 ± 0.392	5% vs 1% (P = 0,001)<br>5% vs 0.25% (P = 0,001)
**Lactate**	7.556 ± 0.803	8.768 ± 1.814	10.552 ± 3.906	n.s.

Table S7: Variations in extracellular metabolites at week 4 expressed as fold change versus acellular medium. Data are presented as average fold change (FC) ± SEM (FC). Significant differences ($P < 0.05$) assessed through one-way-ANOVA with Tukey's HSD for post-hoc testing. n.s. indicates metabolites with no significant changes. Medium was changed every week. (N=3; n=3)

metabolite	fibre-w4-5%	fibre-w4-1%	fibre-w4-0.25%	Significant differences*
**Glucose**	0.004 ± 0.001	0.133 ± 0.085	0.794 ± 0.080	5% vs 0.25% (P = 0,001)<br>1% vs 0.25% (P = 0,001)
**Pyruvate**	0.535 ± 0.017	0.557 ± 0.103	0.986 ± 0.084	5% vs 0.25% (P = 0,015)<br>1% vs 0.25% (P = 0,018)
**Glutamine**	0.624 ± 0.093	0.686 ± 0.059	0.512 ± 0.075	n.s.
**Pyroglutamate**	1.524 ± 0.027	1.537 ± 0.052	1.742 ± 0.284	n.s.
**α-Ketoisocaproate (KIC)**	1.587 ± 0.025	1.481 ± 0.085	1.094 ± 0.194	n.s.
**Alanine**	2.735 ± 0.160	1.718 ± 0.259	0.985 ± 0.210	5% vs 1% (P = 0,035)<br>5% vs 0.25% (P = 0,003)
**Glutamate**	3.704 ± 0.191	2.403 ± 0.153	0.933 ± 0.183	5% vs 1% (P = 0,005)<br>5% vs 0.25% (P = 0,001)<br>1% vs 0.25% (P = 0,003)
**3-Hydroxyisobutyrate (3-HIB)**	3.896 ± 0.175	1.703 ± 0.090	1.749 ± 0.355	5% vs 1% (P = 0,002)<br>5% vs 0.25% (P = 0,002)
**α-Keto-β-methylvalerate (KMV)**	4.115 ± 0.165	4.790 ± 0.263	2.384 ± 0.767	5% vs 0.25% (P = 0,094)<br>1% vs 0.25% (P = 0,028)
**Lactate**	6.410 ± 0.692	14.005 ± 0.943	3.621 ± 1.755	5% vs 1% (P = 0,011)<br>1% vs 0.25% (P = 0,002)
**α-Ketoisovalerate (KIV)**	7.834 ± 0.384	9.361 ± 0.635	3.118 ± 1.361	5% vs 0.25% (P = 0,023)<br>1% vs 0.25% (P = 0,006)
**Formate**	8.921 ± 0.265	6.149 ± 1.170	1.746 ± 0.407	5% vs 0.25% (P = 0,020)

Table S8: Variations in extracellular metabolites at the final time point expressed as fold change versus acellular medium. Data are presented as average fold change (FC) ± SEM (FC). Significant differences (P < 0.05) assessed through one-way-ANOVA with Tukey's HSD for post-hoc testing. n.s. indicates metabolites with no significant changes. Medium was changed every week. (N=3; n=3)

metabolite	fibre-w6-5%	fibre-w6-1%	fibre-w6-0.25%	Significant differences*
**Glucose**	0.004 ± 0.001	0.396 ± 0.083	0.841 ± 0.087	5% vs 1% (P = 0,017)<br>5% vs 0.25% (P = 0,001)<br>1% vs 0.25% (P = 0,009)
**Pyruvate**	0.512 ± 0.041	0.871 ± 0.141	0.763 ± 0.184	n.s.
**Glutamine**	0.593 ± 0.096	0.906 ± 0.050	0.512 ± 0.072	5% vs 1% (P = 0,058)<br>1% vs 0.25% (P = 0,023)
**Acetate**	1.261 ± 0.241	1.156 ± 0.040	1.625 ± 0.122	n.s.
**Pyroglutamate**	1.478 ± 0.012	1.161 ± 0.158	2.119 ± 0.147	5% vs 0.25% (P = 0,025)<br>1% vs 0.25% (P = 0,004)
**α-Ketoisocaproate (KIC)**	1.603 ± 0.096	1.358 ± 0.014	1.178 ± 0.088	5% vs 0.25% (P = 0,017)
**Alanine**	3.617 ± 0.581	1.485 ± 0.101	1.114 ± 0.111	5% vs 1% (P = 0,012)<br>5% vs 0.25% (P = 0,005)
**α-Keto-β-methylvalerate (KMV)**	3.778 ± 0.320	3.684 ± 0.114	2.941 ± 0.738	n.s.
**3-Hydroxyisobutyrate (3-HIB)**	3.840 ± 0.302	1.341 ± 0.125	1.339 ± 0.138	5% vs 1% (P = 0,001)<br>5% vs 0.25% (P = 0,001)
**Glutamate**	4.689 ± 0.565	1.851 ± 0.068	1.168 ± 0.168	5% vs 0.25% (P = 0,020)
**Lactate**	5.609 ± 0.696	10.073 ± 0.812	5.060 ± 2.178	n.s.
**α-Ketoisovalerate (KIV)**	7.867 ± 0.879	6.407 ± 0.093	4.410 ± 1.695	n.s.
**Formate**	9.425 ± 0.550	2.824 ± 0.368	3.654 ± 0.566	5% vs 1% (P = 0,001)<br>5% vs 0.25% (P = 0,001)

Table S9: Average of TEER measurements of DIV-model, mono-culture hCMEC/D3, cultured in 5% FBS, 1% FBS, or 0.25% FBS. Data are presented as mean ± SEM. (n= 6-56, N= 3-4). One-way ANOVA with Dunn's Method for post-hoc testing; p<0.05; * significant against 0.25% FBS.

time	Flocel – 5% FBS [Ohm*cm^2]	Flocel – 1% FBS [Ohm*cm^2]	Flocel – 0.25% FBS [Ohm*cm^2]
week 1	572 ± 40 *	546 ± 29 *	405 ± 28
week 2	530 ± 35	540 ± 40	362 ± 33
week 3	425 ± 42	518 ± 47	365 ± 54
week 4	576 ± 45 *	376 ± 36	304 ± 30
week 5	446 ± 43	486 ± 48	601 ± 55
week 6	623 ± 61	439 ± 71	596 ± 59
week 7	527 ± 65	489 ± 67	621 ± 69

Table S10: Permeability coefficient (PC) of the paracellular marker FD4 from the fibres to the ECS of the DIV-model mono-culture with hCMEC/D3 cultivated in 5% FBS compared to 1% FBS and 0.25% FBS. Permeability coefficients (PC) are given in µm/min and were put into relation to the PC of DIV-model mono-cultures in 5% FBS. (n=3-5, N=3-5). Mean ± SEM; One-way ANOVA. No significances were determined.

FD4	Flocel - 5%		Flocel - 1%		Flocel – 0.25%	
	[µm/min]	[x-fold]	[µm/min]	[x-fold]	[µm/min]	[x-fold]
	11.92	1.00	14.48	1.21	5.79	0.48
	12.01	1.00	6.73	0.56	8.35	0.70
	9.2	0.77	17.04	1.42	7.16	0.60
	8.43	0.70	n.a.	n.a.	n.a.	n.a.
	18.31	1.53	n.a.	n.a.	n.a.	n.a.
**average**	**11.97 ± 1.55**	**1.00 ± 0.13**	**12.75 ± 2.53**	**1.06 ± 0.21**	**7.10 ± 0.60**	**0.59 ± 0.05**

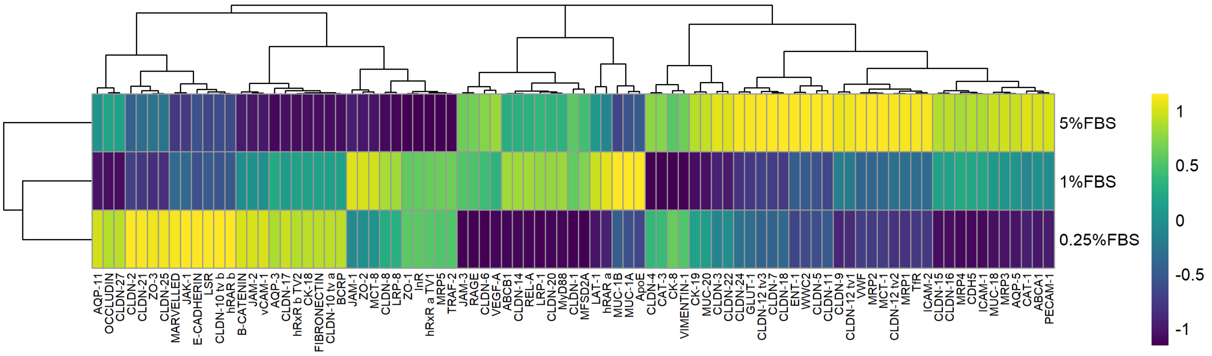

Figure S4: Heat map with hierarchical clustering of targets (Log2(FC)) measured with high-throughput qPCR for samples of hCMEC/D3 cultured in the DIV-model as mono-cultures with 5%, 1% or 0.25% FBS. Complex regulations of mRNA expression levels depending on the serum concentration were determined. Targets were normalized to the housekeeping gene B2M. Log2(FC)-values were annotated to values between -1 to 1. (n=2-5, N=3-5)

Table S11: x-fold values of gene expression changes detected with high throughput qPCR of Flocel mono-cultures cultivated in different serum concentrations of 5%, 1% or 0.25%FBS at the end of the cultivation period. Values were normalized to the housekeeping gene B2M and put into relation to values of the DIV-model mono-cultures in 5%FBS (n=2-5. N=3-5). One-way ANOVA with all pairwise multiple comparison Holm-Sidak in case of non-normality distribution or non-equal variances, p<0.05, * significant against 5% FBS, # significant against Flocel 1% FBS.

target	5%FBS	1%FBS	0.25%FBS
CLDN-1	1.00 ± 0.29	1.03 ± 0.05	0.30 ± 0.007
CLDN-2	1.00 ± 0.31	0.94 ± 0.06	1.48 ± 0.60
CLDN-3	1.00 ± 0.40	0.59 ± 0.14	0.74 ± 0.14
CLDN-4	1.00 ± 0.18	0.67 ± 0.09	0.93 ± 0.02
CLDN-5	1.00 ± 0.33	0.23 ± 0.04	0.23 ± 0.04
CLDN-6	1.00 ± 0.24	0.90 ± 0.19	0.53 ± 0.10
CLDN-7	1.00 ± 0.24	0.44 ± 0.02	0.51 ± 0.11
CLDN-8	1.00 ± 0.61	1.71 ± 0.10	1.45 ± 0.76
CLDN-9	1.00 ± 0.83	0.50 ± 0.00	0.33 ± 0.13
CLDN-10 tv a	1.00 ± 0.64	1.51 ± 0.15	1.95 ± 0.62
CLDN-10 tv b	1.00 ± 0.34	1.04 ± 0.15	1.99 ± 0.44
CLDN-11	1.00 ± 0.16	0.74 ± 0.08	0.76 ± 0.02
CLDN-12 tv1	1.00 ± 0.10	0.73 ± 0.22	0.62 ± 0.06
CLDN-12 tv2	1.00 ± 0.08	0.84 ± 0.02	0.79 ± 0.01
CLDN-12 tv3	1.00 ± 0.05	0.81 ± 0.03	0.84 ± 0.04
CLDN-14	1.00 ± 0.41	1.37 ± 0.97	0.41 ± 0.07
CLDN-15	1.00 ± 0.15	0.74 ± 0.09	0.44 ± 0.05
CLDN-16	1.00 ± 0.07	0.73 ± 0.29	0.43 ± 0.11
CLDN-17	1.00 ± 0.60	1.77 ± 0.03	2.73 ± 0.71
CLDN-18	1.00 ± 0.17	0.55 ± 0.04	0.62 ± 0.17
CLDN-20	1.00 ± 0.51	1.62 ± 0.00	0.20 ± 0.002
CLDN-21	1.00 ± 0.54	0.63 ± 0.22	4.76 ± 1.88
CLDN-22	1.00 ± 0.31	0.38 ± 0.04	0.60 ± 0.002
CLDN-24	1.00 ± 0.40	0.32 ± 0.09	0.44 ± 0.01
CLDN-25	1.00 ± 0.13	0.91 ± 0.08	1.25 ± 0.05
CLDN-27	1.00 ± 0.38	0.08 ± 0.001	4.39 ± 2.09
JAM-1	1.00 ± 0.15	1.43 ± 0.12	1.18 ± 0.30
JAM-2	1.00 ± 0.58	4.23 ± 1.20	17.06 ± 4.53 * (p=0.062)
JAM-3	1.00 ± 0.15	0.80 ± 0.04	0.04 ± 0.01
ZO-1	1.00 ± 0.20	1.73 ± 0.13	1.72 ± 0.21
ZO-2	1.00 ± 0.13	1.58 ± 0.15	1.25 ± 0.04
ZO-3	1.00 ± 0.56	0.55 ± 0.09 * (p=0.071)	4.06 ± 0.53 * (p=0.071)
VWF	1.00 ± 0.42	0.59 ± 0.17	0.53 ± 0.08
GLUT-1	1.00 ± 0.16	0.65 ± 0.02	0.71 ± 0.13
OCCLUDIN	1.00 ± 0.09	0.76 ± 0.09	1.16 ± 0.21
CDH5	1.00 ± 0.42	0.15 ± 0.02	0.004 ± 0.001
ABCB1	1.00 ± 0.19	2.25 ± 0.07 *	0.11 ± 0.02 *#
MRP1	1.00 ± 0.16	0.84 ± 0.11	0.79 ± 0.03
MRP2	1.00 ± 0.22	0.66 ± 0.16	0.60 ± 0.01
MRP3	1.00 ± 0.24	0.70 ± 0.01	0.46 ± 0.08 *
MRP4	1.00 ± 0.12	0.75 ± 0.01	0.40 ± 0.03
MRP5	1.00 ± 0.10	2.28 ± 0.07 *	2.16 ± 0.02 *
BCRP	1.00 ± 0.12	1.45 ± 0.03	1.81 ± 0.14 *
MARVELLED	1.00 ± 0.16	1.07 ± 0.22	1.33 ± 0.04
CAT-1	1.00 ± 0.18	0.53 ± 0.07	0.32 ± 0.02
CAT-3	1.00 ± 0.15	0.59 ± 0.09	0.92 ± 0.04
ENT-1	1.00 ± 0.12	0.77 ± 0.05	0.74 ± 0.07
InR	1.00 ± 0.11	1.36 ± 0.16	1.35 ± 0.02
LAT-1	1.00 ± 0.16	1.62 ± 0.24	0.55 ± 0.02
LRP-1	1.00 ± 0.12	1.08 ± 0.06	0.74 ± 0.06

LRP-8	1.00 ± 0.09	1.47 ± 0.04 * (p=0.067)	1.32 ± 0.04
MCT-1	1.00 ± 0.17	0.79 ± 0.08	0.75 ± 0.08
MCT-8	1.00 ± 0.33	4.57 ± 0.89 * (p=0.062)	2.24 ± 0.31
TfR	1.00 ± 0.12	0.97 ± 0.14	0.96 ± 0.16
VEGF-A	1.00 ± 0.05	0.86 ± 0.17	0.52 ± 0.09
LSR	1.00 ± 0.46	1.33 ± 0.40	18.53 ± 8.83
WWC2	1.00 ± 0.15	0.76 ± 0.10	0.74 ± 0.11
CK-8	1.00 ± 0.13	0.37 ± 0.03	1.03 ± 0.05
CK-18	1.00 ± 0.10	1.63 ± 0.03	2.17 ± 0.04 *
CK-19	1.00 ± 0.53	0.31 ± 0.03	0.66 ± 0.04
AQP-3	1.00 ± 0.37	2.46 ± 0.14	3.73 ± 0.02 *
AQP-5	1.00 ± 0.33	0.54 ± 0.01	0.26 ± 0.01
AQP-11	1.00 ± 0.11	0.75 ± 0.03	1.28 ± 0.09
MUC-1A	1.00 ± 0.16	1.34 ± 0.06	0.99 ± 0.12
MUC-1B	1.00 ± 0.10	1.51 ± 0.33	1.05 ± 0.11
MUC-18	1.00 ± 0.18	0.58 ±0.04	0.29 ± 0.06 *
MUC-20	1.00 ± 0.26	0.31 ±0.09	0.63 ± 0.02
E-CADHERIN	1.00 ± 0.15	1.00 ± 0.14	1.60 ± 0.15
B-CATENIN	1.00 ± 0.16	1.27 ± 0.01	1.66 ± 0.15
VIMENTIN-1	1.00 ± 0.20	0.71 ± 0.07	0.98 ± 0.14
FIBRONECTIN	1.00 ± 0.19	1.36 ± 0.19	1.62 ± 0.16
ABCA1	1.00 ± 0.34	0.62 ± 0.004	0.38 ± 0.08
ApoE	1.00 ± 0.46	1.46 ± 0.10	0.96 ± 0.12
hRAR a	1.00 ± 0.44	1.57 ± 0.27	0.73 ± 0.04
hRAR b	1.00 ± 0.44	1.10 ± 0.01	4.59 ± 1.63
hRxR a TV1	1.00 ± 0.23	2.82 ± 0.23 *	2.69 ± 0.19 *
hRxR b TV2	1.00 ± 0.10	1.16 ±0.18	1.26 ± 0.02
MFSD2A	1.00 ±0.09	1.08 ± 0.03	0.63 ± 0.02 *# (p=0.077, both)
PECAM-1	1.00 ± 0.26	0.61 ± 0.03	0.38 ± 0.04
RAGE	1.00 ± 0.11	0.97 ± 0.09	0.75 ± 0.06
ICAM-1	1.00 ± 0.13	0.95 ± 0.10	0.85 ± 0.13
ICAM-2	1.00 ± 0.10	0.75 ± 0.03	0.69 ± 0.03
JAK-1	1.00 ± 0.10	1.04 ± 0.004	1.24 ± 0.04
MyD88	1.00 ± 0.30	1.24 ± 0.01	0.52 ± 0.02
REL-A	1.00 ± 0.32	1.27 ± 0.17	0.51 ± 0.07
TRAF-2	1.00 ± 0.18	1.21 ± 0.07	1.20 ± 0.01
vCAM-1	1.00 ± 0.37	1.87 ± 0.21	3.54 ± 1.34

Table S12: Average TEER of hCMEC/D3 cultivated in the DIV-model as triple-culture with human primary astrocytes and pericytes (hAP). Average values were normalized to 0 days CK treatment. (n=9-25, N=2-3). Mean ± SEM; Two-way-ANOVA with all pairwise multiple comparison Holm-Sidak in case of non-normality distribution or non-equal variances; $p<0.05$, * sig against 0 days treatment, # sig against 24 h CK treatment.

CK conc	0 days CK treatment		24h CK treatment		14 days CK treatment	
	Ohm*cm^2	[%]	Ohm*cm^2	[%]	Ohm*cm^2	[%]
Control	306 ± 35	100 ± 11	216 ± 47	71 ± 15	269 ± 32	88 ± 11
0.1ng/ml	574 ± 73	100 ± 13	509 ± 54	89 ± 10	436 ± 48	76 ± 8
10ng/ml	868 ± 102	100 ± 12	990 ± 171	114 ± 20	536 ± 71***#**	61 ± 8 ***#**

Table S13: permeability coefficient of paracellular marker FD4 for permeability studies of hCMEC/D3 cultivated in the DIV-model as triple-culture with human primary astrocytes and pericytes. Average values normalized to 0 days CK treatment. (n=3, N=3). Mean ± SEM; Two-way-ANOVA with all pairwise multiple comparison Holm-Sidak in case of non-normality distribution or non-equal variances, no significances were determined.

CK conc	0 days CK treatment		24h CK treatment		14 days CK treatment	
	µm/min	[x-fold]	µm/min	[x-fold]	µm/min	[x-fold]
Control	28.79 ± 10.06	1.00 ± 0.24	26.07 ± 10.49	0.91 ± 0.20	32.43 ± 10.69	1.13 ± 0.25
0.1 ng/ml	27.17 ± 6.91	1.00 ± 0.32	36.18 ± 13.91	1.33 ± 0.21	31.91 ± 14.89	1.17 ± 0.17
10ng/ml	53.01 ± 12.38	1.00 ± 0.35	46.31 ± 16.21	0.87 ± 0.24	51.76 ± 18.52	0.98 ± 0.23

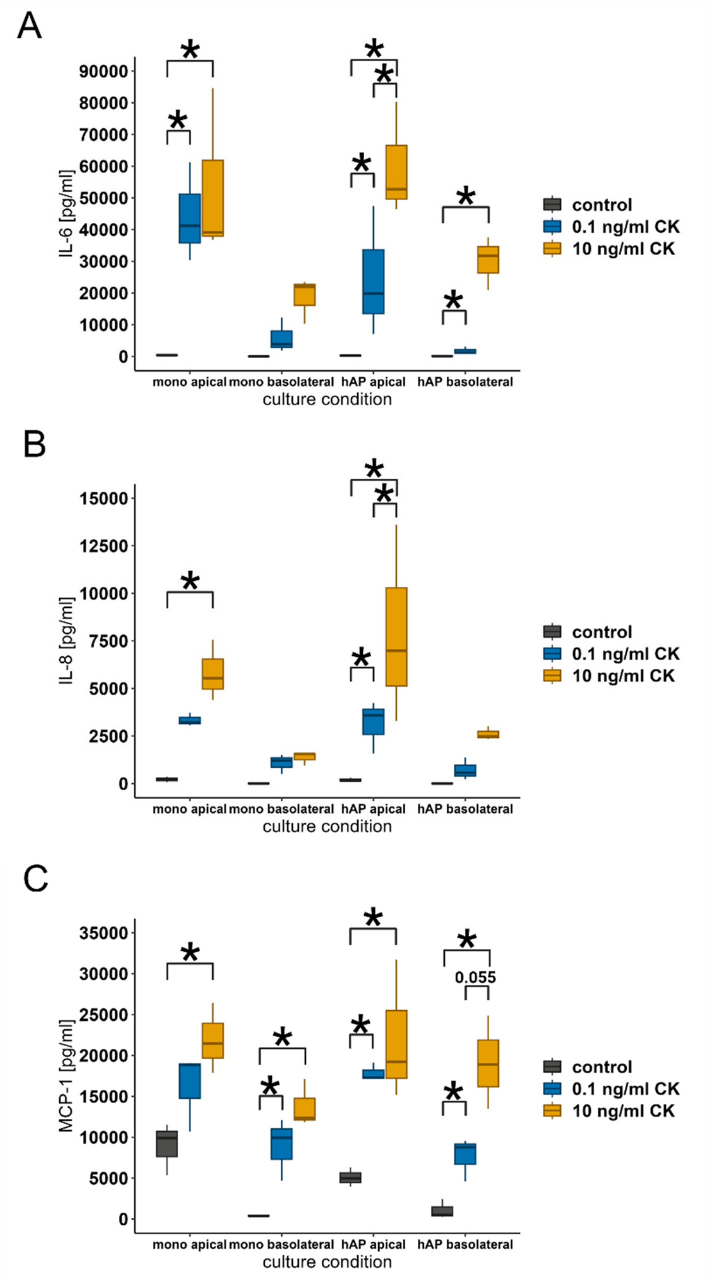

Figure S5: Proof of principle experiment with Transwells®. To determine if hCMEC/D3 are responsive to the CK treatment with 0.1 ng/ml or 10 ng/ml TNF-α, IL-1β and INFγ, hCMEC/D3 were cultivated on Transwells®. Experiments were conducted in mono-cultures and triple-cultures, where hAP were seeded onto the bottom of the plates. CK were added apically, whereas no CK were added basolaterally. As a control, 0.1% BSA was added apically. Samples were collected apically and basolaterally after 24h CK treatment or 24h in 0.1% BSA. Significant changes in the secretion of proinflammatory cytokines IL6 (A), IL-8 (B) and MCP-1 (C) were detected apically and basolaterally (n=3, N=3). Data are presented as mean ± SEM. Two-way-ANOVA with all pairwise multiple comparison Holm-Sidak in case of non-normality distribution or non-equal variances; p<0.05, * significant against 0.1ng/ml or 10 ng/ml CK cocktail.

Table S14: Concentration of proinflammatory cytokines IL-6, IL-8 and MCP-1 in Transwells® cultured with hCMEC/D3 in mono-culture (mono) or in triple-culture with human primary astrocytes and pericytes (hAP) at different time points as a response to treatment with the cytokine cocktail of TNF-α, IL-1β and INF-γ with a concentration of 0.1 ng/ml or 10 ng/ml each or to control treatment with 0.1% BSA. (n=3, N=3); mean ± SEM; Two-way-ANOVA with all pairwise multiple comparison Holm-Sidak in case of non-normality distribution or non-equal variances; $p<0.05$; trending significances are indicated in ( ), $p<0.075$; ***** significant against control, **#** significant against 0.1 ng/ml; **+** significant against mono apical; **$** significant against hAP apical

Cytokine	Culture condition	Transwell® control	Transwell® 0.01 ng/ml	Transwell® 10 ng/ml
IL-6	mono apical	395.2 ± 83.9	44257.4 ± 7377.2 *	53498.3 ± 12704.8 *
	mono basolateral	34.6 ± 20.0	5987.2 ± 2599.4 +	18558.5 ± 3405.7 +
	hAP apical	293.4 ± 58.5	24800.2 ± 9713.9 *	59843.3 ± 8485.8 *#
	hAP basolateral	94.7 ± 23.4	1703.0 ± 566.4 *	30072.2 ± 3968.4 *$
IL-8	mono apical	218.1 ± 68.1	3338.5 ± 161.4 (*)	5828.3 ± 752.5 *
	mono basolateral	17.0 ± 13.9	1075.6 ± 238.4	1363.6 ± 168.7 +
	hAP apical	194.3 ± 47.4	3131.9 ± 652.3 *	7955.3 ± 2464.9 *#
	hAP basolateral	15.0 ± 12.3	725.7 ± 275.8	2610.2 ± 167.7 $
MCP-1	mono apical	8936.4 ± 1502.8	16200.5 ± 2251.3 (*)	21920.6 ± 2017.9 *
	mono basolateral	390.3 ± 29.0	8904.7 ± 1797.8 *	13779.2 ± 1366.7 *
	hAP apical	5075.8 ± 562.1	17845.2 ± 520.9 *	22049.2 ± 4066.4 *
	hAP basolateral	1065.9 ± 556.8	7649.5 ± 1253.2 *$	19082.5 ± 2689.4 *(#)

Table S15: Concentration of proinflammatory cytokines IL-6, IL-8 and MCP-1 in fibres of the DIV-model triple-culture with hCMEC/D3, human primary astrocytes and pericytes at different time points as a response to treatment with the cytokine cocktail of TNF-α, IL-1β and INF-γ with a concentration of 0.1 ng/ml or 10 ng/ml each or to control treatment with 0.1%BSA (n=3, N=3). Data are presented as mean±SEM; Two-way ANOVA with all pairwise multiple comparison Holm-Sidak in case of non-normality distribution or non-equal variances; $p<0.05$; * significant against control, # significant against 0.1 ng/ml; trending signficanies are indicated in ( ), $p<0.075$

Cytokine	Timepoint	Flocel control [pg/ml]	Flocel 0.1 ng/ml CK [pg/ml]	Flocel 10 ng/ml CK [pg/ml]
IL-6	0h fibre	119.0 ± 67.2	941.5 ± 602.4	1375.6 ± 493.5
	6h fibre	84.0 ± 34.3	1195.8 ± 168.4	10560.9 ± 2723.1
	24h fibre	125.0 ± 53.2	5496.8 ± 1668.0	24815.8 ± 5214.1 *(#)
	2 days fibre	217.1 ± 108.8	7125.4 ± 2367.9	30409.6 ± 8817.8 *(#)
	5 days fibre	42.3 ± 19.0	6515.2 ± 1394.6	33411.4 ± 7012.2 *#
	7 days fibre	27.4 ± 22.4	4818.7 ± 2495.5	35713.6 ± 15134.4 *#
	9 days fibre	31.6 ± 25.8	7461.7 ± 3110.8	30833.9 ± 8873.9 *#
	12 days fibre	100.9 ± 49.6	4961.4 ± 1623.7	26588.3 ± 6261.0 *#
	14 days fibre	180.2 ± 96.4	4741.0 ± 1943.0	25359.6 ± 13931.1 *#
IL-8	0h fibre	186.1 ± 102.0	849.0 ± 282.4	1107.8 ± 247.8
	6h fibre	83.7 ± 38.1	1379.6 ± 638.1	1894.6 ± 441.0
	24h fibre	177.1 ± 73.6	2688.4 ± 950.1	5379.3 ± 694.4 *
	2 days fibre	94.5 ± 44.4	1857.3 ± 517.7	3271.7 ± 750.8
	5 days fibre	49.5 ± 31.0	1648.4 ± 314.3	3490.0 ± 294.1
	7 days fibre	31.9 ± 26.0	3534.9 ± 1069.2 (*)	6456.9 ± 1564.3 *(#)
	9 days fibre	2.8 ± 1.6	5482.8 ± 3154.0 *	6851.5 ± 2049.0 *#
	12 days fibre	18.7 ± 7.7	2949.7 ± 1656.0	4595.3 ± 1385.7 *
	14 days fibre	48.9 ± 39.9	2384.7 ± 1061.3	3875.4 ± 716.9
MCP-1	0h fibre	552.8 ± 354.7	3108.8 ± 1596.1	8375.7 ± 2447.2
	6h fibre	140.2 ± 97.4	3500.0 ± 466.7	13276.3 ± 4629.3 *(#)
	24h fibre	225.2 ± 177.4	5190.9 ± 662.4	18337.4 ± 4766.8 *#
	2 days fibre	342.5 ± 140.8	6056.4 ± 785.5	7291.2 ± 404.0
	5 days fibre	244.1 ± 99.7	5249.5 ± 1700.5	7972.3 ± 862.7
	7 days fibre	3.3 ± 2.7	7683.7 ± 1625.2 (*)	22860.0 ± 7802.5 *#
	9 days fibre	191.1 ± 91.5	5746.9 ± 2075.0	8790.3 ± 802.3
	12 days fibre	151.7 ± 63.6	7480.2 ± 927.8	17785.3 ± 4288.2 *#
	14 days fibre	52.5 ± 42.9	8019.8 ± 1018.7	9772.0 ± 3991.5 (*)

Table S16: Concentration of proinflammatory cytokines IL-6, IL-8 and MCP-1 in the ECS of the DIV-model set-ups cultured with hCMEC/D3, human primary astrocytes and pericytes at different time points as a response to treatment with the cytokine cocktail of TNF-α, IL-1β and INF-γ with a concentration of 0.1 ng/ml or 10 ng/ml each or to control treatment with 0.1%BSA. (n=3, N=3); ± SEM; Two-way ANOVA with all pairwise multiple comparison Holm-Sidak in case of non-normality distribution or non-equal variances, $p<0.05$; trending signficanies are indicated in ( ), $p<0.075$; * significant against control, # significant against 0.1 ng/ml

Cytokine	Timepoint	Flocel control [pg/ml]	Flocel 0.1 ng/ml CK [pg/ml]	Flocel 10 ng/ml CK [pg/ml]
IL-6	0h ECS	35.3 ± 28.9	1231.9 ± 492.9	1111.5 ± 337.7
	6h ECS	75.7 ± 32.7	2179.5 ± 589.9	17511.5 ± 5543.1
	24h ECS	60.1 ± 25.2	5056.6 ± 2521.6	28236.8 ± 12500.3 *#
	2 days ECS	112.4 ± 27.9	12407.8 ± 5618.3	27117.3 ± 8814.7 *
	5 days ECS	130.6 ± 90.3	8172.9 ± 2557.0	34758.4 ± 14325.5 *#
	7 days ECS	37.9 ± 31.0	6026.3 ± 3585.1	21221.2 ± 724.6 *
	9 days ECS	31.4 ± 25.6	4961.9 ± 1260.3	32418.6 ± 4006.9 *#
	12 days ECS	62.0 ± 35.3	3725.6 ± 1439.4	19612.1 ± 9641.1
	14 days ECS	342.6 ± 104.2	4429.3 ± 2155.2	22769.4 ± 3866.3 (*#)
IL-8	0h ECS	155.0 ± 31.5	1694.2 ± 679.4	899.0 ± 173.8
	6h ECS	85.1 ± 46.6	1572.0 ± 475.5	3406.8 ± 510.3
	24h ECS	74.6 ± 50.2	2716.3 ± 966.4	5452.1 ± 1730.7 *
	2 days ECS	142.1 ± 50.8	2607.3 ± 1190.5	2866.7 ± 76.9
	5 days ECS	0.0 ± 0.0	3212.2 ± 962.7	4153.1 ± 444.8 *
	7 days ECS	43.0 ± 32.7	2972.9 ± 1573.9	5122.4 ± 1324.0 *
	9 days ECS	72.5 ± 59.2	3417.6 ± 1369.9 (*)	6144.3 ± 1104.7 *
	12 days ECS	202.7 ± 119.2	2783.2 ± 677.9	4584.6 ± 835.7 (*)
	14 days ECS	61.3 ± 43.3	3058.5 ± 1583.6	3467.0 ± 742.7
MCP-1	0h ECS	158.7 ± 38.4	5676.4 ± 1734.0	7883.8 ± 1291.8
	6h ECS	97.1 ± 79.3	4280.4 ± 458.8	17824.0 ± 5318.6 *#
	24h ECS	40.2 ± 25.2	6509.2 ± 974.6	22510.4 ± 7434.2 *#
	2 days ECS	208.6 ± 97.5	6352.7 ± 2327.7	9403.2 ± 967.9
	5 days ECS	255.7 ± 108.2	6555.3 ± 2322.2	9030.5 ± 1627.1
	7 days ECS	1.8 ± 1.5	8391.6 ± 1993.2 (*)	18450.5 ± 6947.0 *#
	9 days ECS	78.0 ± 34.5	5825.6 ± 1855.1	10376.5 ± 827.8 (*)
	12 days ECS	325.0 ± 24.9	6359.8 ± 1119.8	8990.6 ± 892.0
	14 days ECS	92.6 ± 65.4	7552.6 ± 1388.8	12757.5 ± 1216.4 *

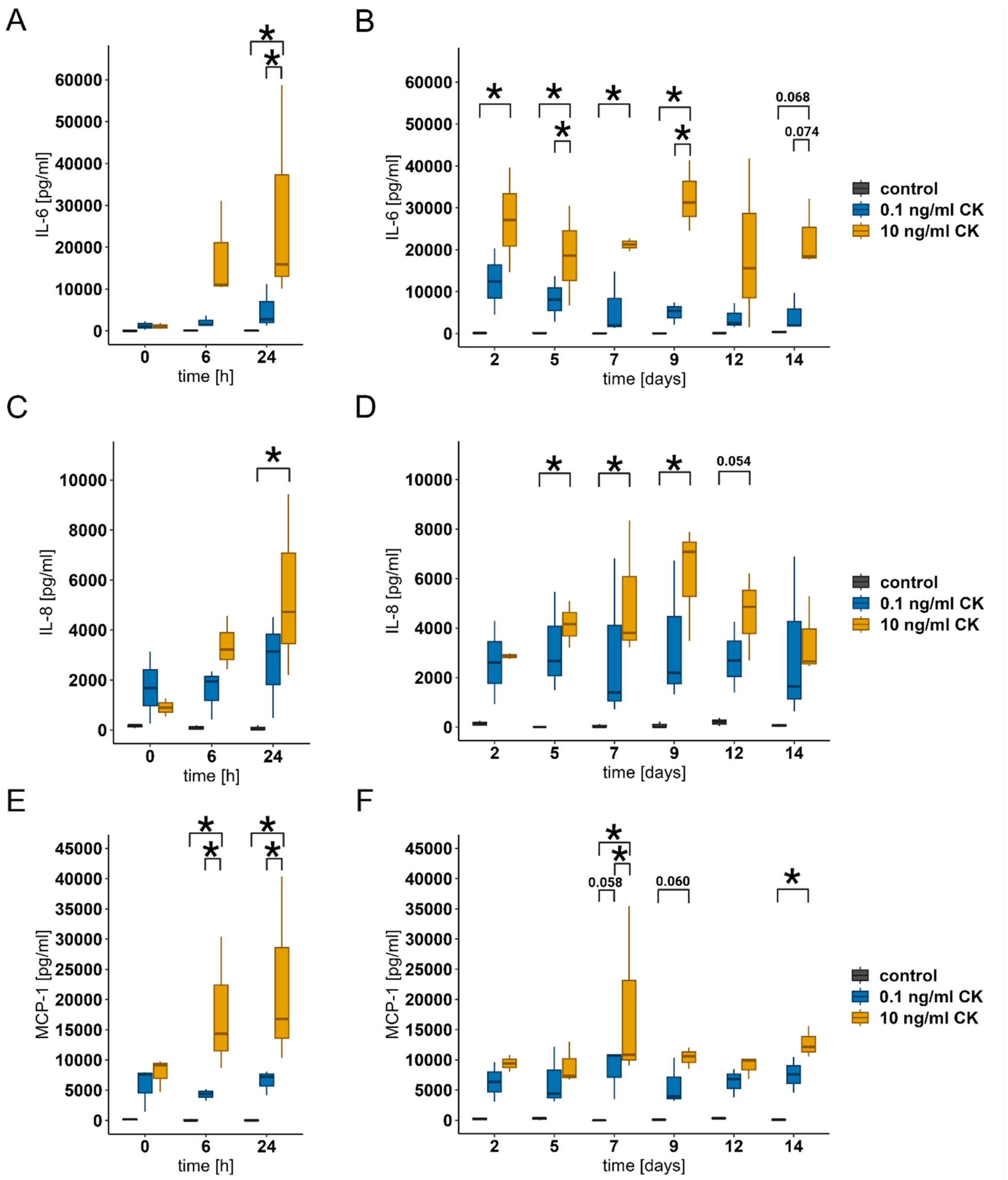

Figure S6: The secretion of proinflammatory cytokines IL-6, IL-8 and MCP-1 by human astrocytes and pericytes (hAP) cultivated in the ECS surrounding the fibres of the DIV-model was determined. A significant increase in IL-6, IL-8 and MCP-1 did occur within 6h and 24 h of CK treatment (A, C, E) and remained increased within the 14 days of CK treatment (B, D, F). hAP were not directly treated with the CK cocktail. Therefore, any changes in the concentration of CKs in the ECS were either caused by diffusion across the cell layer within the fibres or by proinflammatory factors secreted into the ECS by hCMEC/D3 cultured in the fibres as a response to the CK treatment. It should be noted that medium changes including the addition of fresh CKs were performed after the permeability study with the paracellular marker FD4 after 24h CK treatment as well as on day 7 after the start of CK treatment. (n=2-3, N=2-3). Data are presented as mean ± SEM. Two-way-ANOVA with all pairwise multiple comparison Holm-Sidak in case of non-normality distribution or non-equal variances; $p<0.05$,* significant against 0.1 ng/ml or 10 ng/ml CK cocktail.

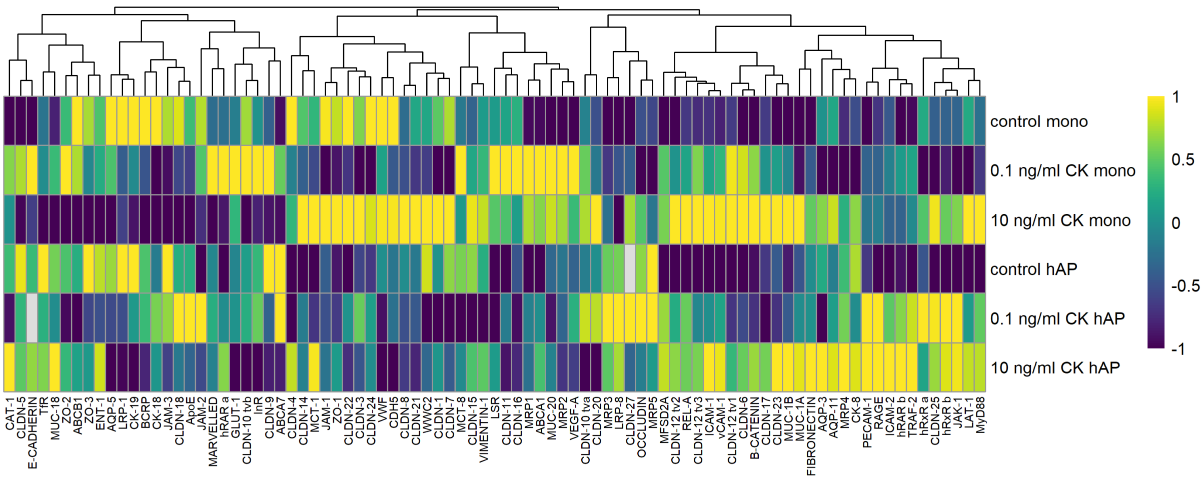

Figure S7: Heat map with hierarchical clustering of targets (Log2(FC)) measured with high-throughput qPCR for samples of hCMEC/D3 cultured in Transwells® as mono-culture (mono) or triple-culture with human primary astrocytes and pericytes (hAP). Both, mono- and triple-cultures were treated with 0.1 ng/ml or 10 ng/ml TNF-α, IL-1β and INFγ, or 0.1% BSA as control for 24 hours. Complex regulations of mRNA expression levels depending on the CK concentration as well as on the culture condition as mono- or triple-culture were determined. Targets were normalized to the housekeeping gene GAPDH. Log2(FC)-values were rescaled to a standardized range of -1 to 1. (n=2-3, N=2-3).

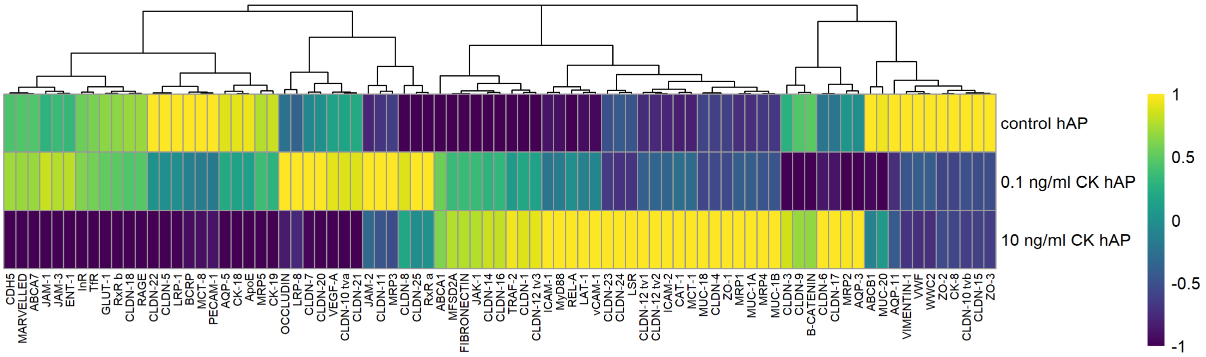

Figure S8: Heat map with hierarchical clustering of targets (Log2(FC)) measured with high-throughput qPCR for samples of hCMEC/D3 cultured in Transwells® as triple-culture with human primary astrocytes and pericytes (hAP). Triple-cultures were treated with 0.1 ng/ml or 10 ng/ml TNF-α, IL-1β and INFγ, or 0.1% BSA as control for 24 hours by adding the CK cocktail apically. Complex regulations of mRNA expression levels depending on the CK concentration as well as on the culture condition as mono- or triple-culture were determined. Targets were normalized to the housekeeping gene GAPDH. Log2(FC)-values were rescaled to a standardized range of -1 to 1. (n=2-3, N=2-3).

Table S17: x-fold values of high throughput qPCR of hCMEC/D3 cultivated on Transwells® in mono-culture or in co-culture with human astrocytes and pericytes (hAP) for 24 hours with 0.1 ng/ml or 10 ng/ml CK cocktail consisting of TNF-a, IL-1b or IFN-g each. Values were normalized to the housekeeping gene GAPDH and put into relation to values of mono-cultures treated with 0.1%BSA for 24 hours (control) (n=3. N=2-3). Two-way-ANOVA with all pairwise multiple comparison Holm-Sidak in case of non-normality distribution or non-equal variances; p<0.05, * significant against mono control, **#** significant against 0.1 ng/ml CK mono, **+** significant against 10 ng/ml CK mono, **§** significant against control hAP, **&** significant against 0.1 ng/ml CK hAP

target	control mono	0.1 ng/ml CK mono	10 ng/ml CK mono	control hAP	0.1 ng/ml CK hAP	10 ng/ml CK hAP
CLDN-1	1.00 ± 0.51	0.36 ± 0.16	2.00 ± 0.83	0.75 ± 0.51	0.39 ± 0.01	0.76 ± 0.08
CLDN-3	1.00 ± 0.51	0.14 ± 0.09	2.07 ± 0.56 **#**	0.31 ± 0.15	0.93 ± 0.21 **#** (0.060)	0.35 ± 0.02 **+**
CLDN-4	1.00 ± 0.60	0.57 ± 0.04	0.68 ± 0.26	0.37 ± 0.16	0.39 ± 0.19	0.83 ± 0.00003
CLDN-5	1.00 ± 0.69	2.37 ± 0.27 *	1.23 ± 0.14 *	2.52 ± 0.29 *	1.99 ± 0.003	2.14 ± 0.002 **+**
CLDN-6	1.00 ± 0.67	2.31 ± 1.12	2.75 ± 0.66	1.03 ± 0.39	1.25 ± 0.19	2.01 ± 0.20
CLDN-7	1.00 ± 0.53	0.39 ± 0.11	1.26 ± 0.22	0.96 ± 0.37	0.43 ± 0.11	0.56 ± 0.10
CLDN-8	1.00 ± 0.50	0.84 ± 0.26	6.47 ± 1.80	1.24 ± 0.59	0.84 ± 0.22	0.75 ± 0.05
CLDN-9	1.00 ± 0.54	64.00 ± 44.63	0.32 ± 0.21	27.47 ± 11.46	1.04 ± 0.45	0.37 ± 0.08
CLDN-10 tva	1.00 ± 0.65	2.01 ± 0.49	2.59 ± 0.30 *	0.96 ± 0.10	2.80 ± 0.44 **§**	0.15 ± 0.02 **+&**
CLDN-10 tvb	1.00 ± 0.65	1.55 ± 0.41	0.26 ± 0.08	0.63 ± 0.07	0.70 ± 0.17	0.21 ± 0.00
CLDN-11	1.00 ± 0.21	1.96 ± 0.11 *	1.13 ± 0.12 **#**	0.54 ± 0.02 * (0.053)	0.86 ± 0.16 **#**	0.61 ± 0.10 **+**
CLDN-12 tv1	1.00 ± 0.11	1.54 ± 0.32	1.68 ± 0.19	0.86 ± 0.01	0.99 ± 0.02	1.30 ± 0.02
CLDN-12 tv2	1.00 ± 0.25	1.46 ± 0.35	2.15 ± 0.15	1.07 ± 0.02	1.54 ± 0.26	1.74 ± 0.23
CLDN-12 tv3	1.00 ± 0.49	1.69 ± 0.32	2.10 ± 0.05 *	0.74 ± 0.15	1.30 ± 0.09	1.68 ± 0.13 **§** (0.062)
CLDN-14	1.00 ± 0.05	0.28 ± 0.06	6.06 ± 2.60	0.06 ± 0.01	0.39 ± 0.11	0.42 ± 0.11
CLDN-15	1.00 ± 0.28	1.25 ± 0.56	1.67 ± 0.28	1.50 ± 0.21	0.55 ± 0.18	1.37 ± 0.20
CLDN-16	1.00 ± 0.33	2.90 ± 1.30	0.89 ± 0.02	0.59 ± 0.25	0.43 ± 0.12	0.48 ± 0.12
CLDN-17	1.00 ± 0.45	1.50 ± 0.76	4.04 ± 1.05	0.99 ± 0.28	0.82 ± 0.45	2.07 ± 0.74
CLDN-18	1.00 ± 0.43	0.65 ± 0.21	0.48 ± 0.13	0.83 ± 0.26	1.10 ± 0.30	0.73 ± 0.17
CLDN-20	1.00 ± 0.46	1.28 ± 0.81	4.60 ± 1.66	1.49 ± 0.68	2.84 ± 0.64	0.45 ± 0.08
CLDN-22	1.00 ± 0.05	0.42 ± 0.01	0.89 ± 0.07	0.36 ± 0.07	0.57 ± 0.31	0.41 ± 0.04
CLDN-23	1.00 ± 0.81	7.15 ± 4.48	31.38 ± 0.95 *#	3.27 ± 0.63	2.20 ± 0.23	31.92 ± 1.83 **§&**
CLDN-24	1.00 ± 0.08	0.15 ± 0.03 *	0.77 ± 0.26 **#**	0.26 ± 0.08 *	0.45 ± 0.03	0.41 ± 0.03
CLDN-21	1.00 ± 0.25	0.48 ± 0.29	4.32 ± 1.02	0.72 ± 0.09	0.59 ± 0.05	0.44 ± 0.10
JAM-1	1.00 ± 0.12	0.50 ± 0.08	1.15 ± 0.30	0.65 ± 0.20	0.63 ± 0.13	0.69 ± 0.03
JAM-2	1.00 ± 0.12	0.81 ± 0.24	0.38 ± 0.01	0.27 ± 0.17	1.41 ± 0.74	0.43 ± 0.11
JAM-3	1.00 ± 0.07	0.59 ± 0.16	0.55 ± 0.15	1.17 ± 0.06	0.98 ± 0.34	0.65 ± 0.22
ZO1	1.00 ± 0.49	0.31 ± 0.13 *	1.63 ± 0.13 *#	0.32 ± 0.14 *	0.37 ± 0.08	0.61 ± 0.11 **+**
ZO2	1.00 ± 0.69	1.27 ± 0.57	0.64 ± 0.22	1.02 ± 0.04	0.72 ± 0.26	0.99 ± 0.03
ZO3	1.00 ± 0.07	0.45 ± 0.01	0.11 ± 0.004	1.87 ± 0.79	0.28 ± 0.05	0.52 ± 0.01
VWF	1.00 ± 0.35	0.54 ± 0.22	0.88 ± 0.30	0.49 ± 0.26	0.28 ± 0.05	0.21 ± 0.03
GLUT-1	1.00 ± 0.51	1.34 ± 0.06	1.08 ± 0.02	1.01 ± 0.39	1.03 ± 0.43	0.77 ± 0.30
Occludin	1.00 ± 0.74	0.45 ± 0.20	1.96 ± 0.40 **#** (0.071)	2.26 ± 0.26 *	3.30 ± 0.46 **#**	1.29 ± 0.08 **&**
CDH5	1.00 ± 0.75	0.53 ± 0.16	0.87 ± 0.31	0.58 ± 0.05	0.57 ± 0.08	0.38 ± 0.11
ABCB1	1.00 ± 0.48	0.88 ± 0.36	0.50 ± 0.19	0.77 ± 0.08	0.55 ± 0.05	0.75 ± 0.09
MRP1	1.00 ± 0.48	2.81 ± 1.50	2.00 ± 0.47	0.98 ± 0.28	1.40 ± 0.06	1.33 ± 0.06
MRP2	1.00 ± 0.45	3.10 ± 0.38	2.13 ± 0.39	1.44 ± 0.20	1.37 ± 0.21	1.19 ± 0.03
MRP3	1.00 ± 0.52	1.31 ± 0.56	1.51 ± 0.13	1.96 ± 0.06	2.58 ± 0.11 **#** (0.072)	1.93 ± 0.13
MRP4	1.00 ± 0.43	0.80 ± 0.01	1.32 ± 0.14	1.36 ± 0.10 * (0.056)	1.43 ± 0.11 **#**	1.67 ± 0.10
MRP5	1.00 ± 0.31	0.78 ± 0.12	1.10 ± 0.45	1.56 ± 0.24	1.58 ± 0.31	1.20 ± 0.21
BCRP	1.00 ± 0.34	0.50 ± 0.05 *	0.40 ± 0.06 *	0.68 ± 0.05 *	0.66 ± 0.07	0.45 ± 0.02
Marvelled	1.00 ± 0.80	2.36 ± 1.24	0.60 ± 0.13	1.10 ± 0.05	1.27 ± 0.01	1.08 ± 0.12
CAT1	1.00 ± 0.36	1.59 ± 0.31	1.38 ± 0.11	1.49 ± 0.25	1.12 ± 0.47	1.84 ± 0.27
ENT1	1.00 ± 0.36	0.97 ± 0.31	0.71 ± 0.27	1.06 ± 0.09	0.86 ± 0.23	1.07 ± 0.20
InR	1.00 ± 0.56	1.69 ± 0.30	0.71 ± 0.28	1.12 ± 0.20	1.22 ± 0.23	0.55 ± 0.27

LAT1	1.00 ± 0.28	0.37 ± 0.03	2.18 ± 0.40 **#** ***** (0.066)	0.33 ± 0.13	0.92 ± 0.16	1.60 ± 0.23
LRP1	1.00 ± 0.57	0.57 ± 0.21	0.53 ± 0.08	1.01 ± 0.05	0.54 ± 0.15	0.47 ± 0.08
LRP8	1.00 ± 0.45	1.19 ± 0.44	1.01 ± 0.41	1.62 ± 0.20	2.03 ± 0.24	1.70 ± 0.28
MCT1	1.00 ± 0.29	0.67 ± 0.04	1.53 ± 0.12 **#** (0.076)	0.48 ± 0.23	0.63 ± 0.25	1.36 ± 0.18 **§** (0.072)
MCT8	1.00 ± 0.41	2.87 ± 1.21	1.20 ± 0.56	1.75 ± 0.45	0.34 ± 0.16	1.11 ± 0.45
TfR	1.00 ± 0.30	1.05 ± 0.05	0.67 ± 0.25	1.42 ± 0.15	1.13 ± 0.39	1.24 ± 0.19
VEGF-A tv4	1.00 ± 0.46	3.51 ± 0.50	2.00 ± 0.97	1.47 ± 0.30	1.93 ± 0.22	1.31 ± 0.04
LSR	1.00 ± 0.15	3.54 ± 2.40	1.38 ± 0.08	0.39 ± 0.15	0.33 ± 0.15	0.99 ± 0.07
WWC2	1.00 ± 0.51	0.72 ± 0.21	1.56 ± 0.12	1.30 ± 0.19	0.53 ± 0.25	0.81 ± 0.29
CK8	1.00 ± 0.81	0.51 ± 0.26	1.98 ± 0.29	2.09 ± 0.59	1.33 ± 0.90	2.26 ± 0.17
CK18	1.00 ± 0.80	0.46 ± 0.02	0.46 ± 0.12	0.59 ± 0.08	0.75 ± 0.29	0.64 ± 0.12
CK19	1.00 ± 0.78	0.46 ± 0.02	0.27 ± 0.06 *****	0.95 ± 0.21	0.50 ± 0.20	0.20 ± 0.06 **§**
AQP3	1.00 ± 0.69	0.51 ± 0.17	1.41 ± 0.10	1.11 ± 0.12	0.56 ± 0.34	2.13 ± 0.34 **&**
AQP5	1.00 ± 0.80	0.37 ± 0.08 *****	0.09 ± 0.04 ***#**	0.48 ± 0.05 *****	0.14 ± 0.07 **#§**	0.09 ± 0.04 **§**
AQP11	1.00 ± 0.69	0.54 ± 0.24	1.21 ± 0.16	0.90 ± 0.39	0.98 ± 0.63	1.23 ± 0.17
MUC1A	1.00 ± 0.69	1.07 ± 0.35	6.29 ± 0.41 ***#**	1.49 ± 0.48	2.07 ± 1.08	4.17 ± 1.27
MUC1B	1.00 ± 0.69	2.02 ± 0.69	5.89 ± 0.35 *****	1.17 ± 0.32	1.59 ± 0.85	4.66 ± 1.98
MUC18	1.00 ± 0.69	1.63 ± 0.48	0.77 ± 0.25	1.79 ± 0.001	1.39 ± 0.78	2.47 ± 0.15
MUC20	1.00 ± 0.81	3.71 ± 1.03	2.60 ± 0.21	1.43 ± 0.79	1.08 ± 0.72	1.61 ± 0.31
e-cadherin	1.00 ± 0.20	7.45 ± 4.27	1.92 ± 0.11	4.30 ± 2.57	0.00 ± 0.00	5.54 ± 0.00
b-catenin	1.00 ± 0.80	1.52 ± 0.63	1.80 ± 0.07	1.02 ± 0.28	1.07 ± 0.56	1.54 ± 0.08
vimentin-1	1.00 ± 0.69	1.45 ± 0.65	3.00 ± 0.02 ***** (0.051)	1.29 ± 0.32	0.04 ± 0.02 **#** (0.068)	1.82 ± 0.07
fibronectin	1.00 ± 0.80	1.66 ± 0.65	2.58 ± 0.04	2.01 ± 0.43	2.04 ± 1.06	4.01 ± 0.51
CLDN-25	1.00 ± 0.81	0.58 ± 0.07	2.38 ± 0.49	0.66 ± 0.15	2.43 ± 1.12	2.01 ± 0.52
CLDN-27	1.00 ± 0.82	3.79 ± 0.92	21.08 ± 10.75	0.00 ± 0.00	49.82 ± 0.00	4.02 ± 0.00
ABCA1	1.00 ± 0.78	4.68 ± 0.63 *****	2.96 ± 0.13 ***#**	0.76 ± 0.06	1.91 ± 0.58 **#**	2.56 ± 0.08
ABCA7	1.00 ± 0.80	2.31 ± 1.00	1.01 ± 0.00	3.08 ± 0.36	3.18 ± 1.56	1.06 ± 0.17
ApoE	1.00 ± 0.80	0.72 ± 0.01	0.58 ± 0.12	0.97 ± 0.24	1.52 ± 0.26	0.79 ± 0.30
hRAR a TV1-4	1.00 ± 0.80	2.96 ± 0.64	0.71 ± 0.00	0.63 ± 0.00	1.16 ± 0.02	1.68 ± 0.56
hRAR b Tvb	1.00 ± 0.81	3.57 ± 1.99	2.11 ± 0.03	1.17 ± 0.47	6.49 ± 4.46	19.14 ± 0.00
hRxR a TV1	1.00 ± 0.02	0.27 ± 0.03	1.19 ± 0.44	0.62 ± 0.02	2.19 ± 0.82	0.97 ± 0.10
hRxR b TV2	1.00 ± 0.80	0.83 ± 0.18	1.43 ± 0.13	0.67 ± 0.24	1.82 ± 0.37	1.64 ± 0.32
Mfsd2a	1.00 ± 0.80	6.36 ± 1.46	7.17 ± 1.83	1.24 ± 0.39	7.87 ± 1.04	8.92 ± 3.80
Pecam-1	1.00 ± 0.80	1.39 ± 0.63	1.47 ± 0.04	1.23 ± 0.63	2.78 ± 0.41	2.48 ± 0.29
RAGE	1.00 ± 0.80	1.09 ± 0.06	1.17 ± 0.13	0.87 ± 0.28	1.79 ± 0.16	1.63 ± 0.23
ICAM1 tvb	1.00 ± 0.79	2.03 ± 0.21	4.07 ± 0.49 ***** (0.075)	0.53 ± 0.16	1.76 ± 0.09	4.15 ± 1.73 **§** (0.058)
ICAM2 tva	1.00 ± 0.79	1.14 ± 0.03	1.04 ± 0.23	0.72 ± 0.18	1.47 ± 0.10	2.39 ± 0.70
JAK1 tvb	1.00 ± 0.81	0.72 ± 0.21	1.57 ± 0.15	0.67 ± 0.25	1.94 ± 0.34 **#§**	1.85 ± 0.18 **§**
MyD88 tvb	1.00 ± 0.81	0.71 ± 0.22	3.05 ± 0.51 ***** (0.053) **#** (0.053)	0.36 ± 0.16	1.84 ± 0.64	2.33 ± 0.51
RelA	1.00 ± 0.81	2.91 ± 2.21	9.15 ± 2.83	0.29 ± 0.15	4.58 ± 2.39	4.66 ± 1.16
TRAF-2 tvc	1.00 ± 0.80	1.79 ± 0.45	1.59 ± 0.35	0.84 ± 0.25	2.50 ± 0.40	3.17 ± 0.62 **§** **+** (0.051)
vCAM1 tva	1.00 ± 0.28	7.66 ± 4.46	25.63 ± 5.80 ***** **#** (0.071)	0.25 ± 0.08	5.90 ± 2.09	24.52 ± 7.43 **§**

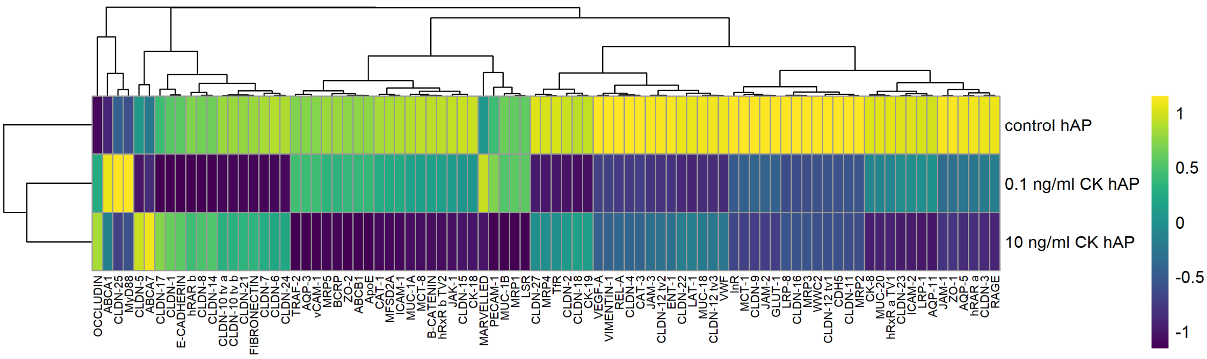

Figure S9: Heat map with hierarchical clustering of targets (Log2(FC)) measured with high-throughput qPCR for samples of hCMEC/D3 cultured in the DIV-model as triple-cultures after 14 days treatment with 0.1 ng/ml or 10 ng/ml TNFα, IL-1β and INFγ, or 0.1% BSA as control. Complex regulations of mRNA expression levels depending on the CK concentration were determined. Targets were normalized to the housekeeping gene GAPDH. Log2(FC)-values were rescaled to values between -1 to 1. (n=2-3, N=2-3).

Table S18: x-fold values of high throughput qPCR of hCMEC/D3 cultured in the DIV-model as triple-cultures cultivated with a serum concentration of 0.25%FBS. Values were normalized to the housekeeping gene GAPDH and put into relation to values of the DIV-model triple-cultures treated with 0.1%BSA for 14 days (control) (n=2-3. N=2-3). One-way ANOVA with all pairwise multiple comparison Holm-Sidak in case of non-normality distribution or non-equal variances, p<0.05, * significant against control, p<0.05; # significant against Flocel 0.1 ng/ml.

target	control	0.1 ng/ml CK	10 ng/ml CK
CLDN-1	1.00 ± 0.66	0.28 ± 0.06	1.04 ± 0.11
CLDN-2	1.00 ± 0.50	0.11 ± 0.02	0.38 ± 0.17
CLDN-3	1.00 ± 0.00	0.17 ± 0.01	0.06 ± 0.02
CLDN-4	1.00 ± 0.42	0.22 ± 0.10	0.29 ± 0.01
CLDN-5	1.00 ± 0.13	0.45 ± 0.15 *(0.052)	1.86 ± 0.04 *#
CLDN-6	1.00 ± 0.02	0.09 ± 0.02 *(0.067)	0.45 ± 0.03
CLDN-7	1.00 ± 0.00	0.32 ± 0.13	0.72 ± 0.12
CLDN-8	1.00 ± 0.34	0.06 ± 0.01	0.80 ± 0.43
CLDN-9	1.00 ± 0.63	0.01 ± 0.01	0.004 ± 0.0001
CLDN-10 tv a	1.00 ± 0.35	0.02 ± 0.003	0.39 ± 0.03
CLDN-10 tv b	1.00 ± 0.35	0.05 ± 0.003	0.47 ± 0.24
CLDN-11	1.00 ± 0.61	0.48 ± 0.25	0.47 ± 0.09
CLDN-12 tv1	1.00 ± 0.71	0.14 ± 0.004	0.14 ± 0.03
CLDN-12 tv2	1.00 ± 0.61	0.23 ± 0.05	0.34 ± 0.05
CLDN-12 tv3	1.00 ± 0.63	0.35 ± 0.01	0.49 ± 0.001
CLDN-14	1.00 ± 0.68	0.04 ± 0.02	0.83 ± 0.51
CLDN-15	1.00 ± 0.55	0.48 ± 0.05	0.20 ± 0.07
CLDN-16	1.00 ± 0.59	0.19 ± 0.12	0.20 ± 0.06
CLDN-17	1.00 ± 0.31	0.10 ± 0.02	1.57 ± 0.75
CLDN-18	1.00 ± 0.05	0.33 ± 0.11 *	0.65 ± 0.06
CLDN-22	1.00 ± 0.13	0.09 ± 0.01 *(0.062)	0.22 ± 0.04
CLDN-23	1.00 ± 0.23	0.04 ± 0.001	0.002 ± 0.001
CLDN-24	1.00 ± 0.01	0.04 ± 0.004	0.36 ± 0.21
CLDN-21	1.00 ±0.31	0.09 ± 0.02	0.58 ± 0.23
JAM-1	1.00 ± 0.65	0.38 ± 0.10	0.28 ± 0.05
JAM-2	1.00 ± 0.00	0.07 ± 0.02	0.04 ± 0.01
JAM-3	1.00 ± 0.62	0.06 ± 0.02	0.12 ± 0.01
ZO-1	1.00 ± 0.57	0.37 ± 0.01	0.26 ± 0.04
ZO-2	1.00 ± 0.68	0.81 ± 0.01	0.33 ± 0.06
VWF	1.00 ± 0.60	0.05 ± 0.004 *(0.076)	0.12 ± 0.03
GLUT-1	1.00 ± 0.60	0.33 ± 0.002	0.27 ± 0.05
OCCLUDIN	1.00 ± 0.00	3.98 ± 1.25	7.03 ± 2.39
CDH5	1.00 ± 0.00	0.15 ± 0.02	0.15 ± 0.002
ABCB1	1.00 ± 0.65	0.78 ± 0.21	0.31 ± 0.01
MRP1	1.00 ± 0.62	0.98 ± 0.40	0.24 ± 0.001
MRP2	1.00 ± 0.01	0.04 ± 0.004	0.04 ± 0.005
MRP3	1.00 ± 0.65	0.17 ± 0.02	0.18 ± 0.03
MRP4	1.00 ± 0.65	0.24 ± 0.12	0.49 ± 0.09
MRP5	1.00 ± 0.65	0.67 ± 0.02	0.19 ± 0.01
BCRP	1.00 ± 0.15	0.64 ± 0.15	0.17 ± 0.02 *
MARVELLED	1.00 ± 0.63	1.66 ± 0.58	0.54 ± 0.10

CAT-1	1.00 ± 0.54	0.89 ± 0.03	0.68 ± 0.01
CAT-3	1.00 ± 0.50	0.12 ± 0.06	0.17 ± 0.05
ENT-1	1.00 ± 0.66	0.25 ± 0.01	0.36 ± 0.05
InR	1.00 ± 0.59	0.60 ± 0.12	0.57 ± 0.04
LAT-1	1.00 ± 0.65	0.15 ± 0.03	0.30 ± 0.02
LRP-1	1.00 ± 0.55	0.23 ± 0.10	0.05 ± 0.01
LRP-8	1.00 ± 0.63	0.29 ± 0.06	0.31 ± 0.06
MCT-1	1.00 ± 0.61	0.40 ± 0.06	0.34 ± 0.08
MCT-8	1.00 ± 0.65	0.20 ± 0.002	0.02 ± 0.002
TfR	1.00 ± 0.51	0.46 ± 0.10	0.68 ± 0.11
VEGF-A	1.00 ± 0.64	0.37 ± 0.02	0.43 ± 0.01
LSR	1.00 ± 0.00	0.97 ± 0.03	0.16 ± 0.03
WWC2	1.00 ± 0.59	0.47 ± 0.03	0.47 ± 0.01
CK-8	1.00 ± 0.51	0.57 ± 0.18	0.36 ± 0.11
CK-18	1.00 ± 0.60	0.57 ± 0.04	0.29 ± 0.06
CK-19	1.00 ± 0.54	0.31 ± 0.10	0.62 ± 0.05
AQP-3	1.00 ± 0.69	0.54 ± 0.17	0.02 ± 0.01
AQP-5	1.00 ± 0.00	0.02 ± 0.0002 *****	0.003 ± 0.0002 ***#**
AQP-11	1.00 ± 0.68	0.25 ± 0.12	0.06 ± 0.01
MUC-1A	1.00 ± 0.70	0.50 ± 0.10	0.15 ± 0.02
MUC-1B	1.00 ± 0.62	0.95 ± 0.43	0.50 ± 0.11
MUC-18	1.00 ± 0.68	0.65 ± 0.06	0.75 ± 0.12
MUC-20	1.00 ± 0.41	0.10 ± 0.01	0.02 ± 0.000024
E-CADHERIN	1.00 ± 0.00	0.53 ± 0.22	0.00 ± 0.00
B-CATENIN	1.00 ± 0.64	0.51 ± 0.20	0.18 ± 0.03
VIMENTIN-1	1.00 ± 0.66	0.39 ± 0.05	0.45 ± 0.02
FIBRONECTIN	1.00 ± 0.16	0.35 ± 0.02	0.78 ± 0.21
CLDN-25	1.00 ± 0.60	2.20 ± 0.12	0.88 ± 0.002
CLDN-27	1.00 ± 0.27	0.005 ± 0.002	0.06 ± 0.02
ABCA1	1.00 ± 0.00	4.51 ± 2.01	1.78 ± 0.09
ABCA7	1.00 ± 0.17	0.95 ± 0.16	1.11 ± 0.32
ApoE	1.00 ± 0.69	0.51 ± 0.18	0.03 ± 0.01
hRAR a	1.00 ± 0.71	0.24 ± 0.01	0.12 ± 0.001
hRAR b	1.00 ± 0.22	0.06 ± 0.01	0.66 ± 0.18
hRxR a TV1	1.00 ± 0.68	0.17 ± 0.004	0.05 ± 0.002
hRxR b TV2	1.00 ± 0.63	0.44 ± 0.11	0.12 ± 0.01
MFSD2A	1.00 ±0.69	0.86 ± 0.02	0.62 ± 0.10
PECAM-1	1.00 ± 0.66	1.18 ± 0.04	0.38 ± 0.04
RAGE	1.00 ± 0.18	0.63 ± 0.19	0.47 ± 0.13
ICAM-1	1.00 ± 0.57	0.79 ± 0.08	0.48 ± 0.01
ICAM-2	1.00 ± 0.59	0.32 ± 0.04	0.11 ± 0.01
JAK-1	1.00 ± 0.64	0.58 ± 0.20	0.27 ± 0.02
MyD88	1.00 ± 0.68	2.79 ± 0.50	1.02 ± 0.04
REL-A	1.00 ± 0.66	0.27 ± 0.02	0.35 ± 0.02
TRAF-2	1.00 ± 0.61	0.84 ± 0.18	0.27 ± 0.002
vCAM-1	1.00 ± 0.69	0.63 ± 0.40	0.06 ± 0.01

Table S19: Comparison of the gene expression of inflammatory markersin hCMEC/D3 in control triple-cultures of the Transwell®system to the Flocel system. Date were normalized to the housekeeping gene GAPDH. Analysis with Student's t-test did not reveal any significant regulations. (n=2-3. N=2-3).

	PECAM-1	RAGE	ICAM-1	ICAM-2	JAK-1	MyD88	REL-A	TRAF-2	vCAM-1
Flocel triple-culture control	10.5 ± 1.8	8.5 ± 0.4	1.5 ± 1.1	4.5 ±1.1	5.0 ± 1.4	13.0 ± 2.1	10.5 ± 1.8	5.0 ± 1.4	8.0 ± 2.1
Transwell® triple-culture control	11.5 ± 0.4	9.0 ± 0.0	3.0 ± 0.0	5.5 ±0.4	7.0 ± 0.0	14.0 ± 0.0	15.5 ± 0.4	5.5 ± 0.4	14.0 ± 0.0

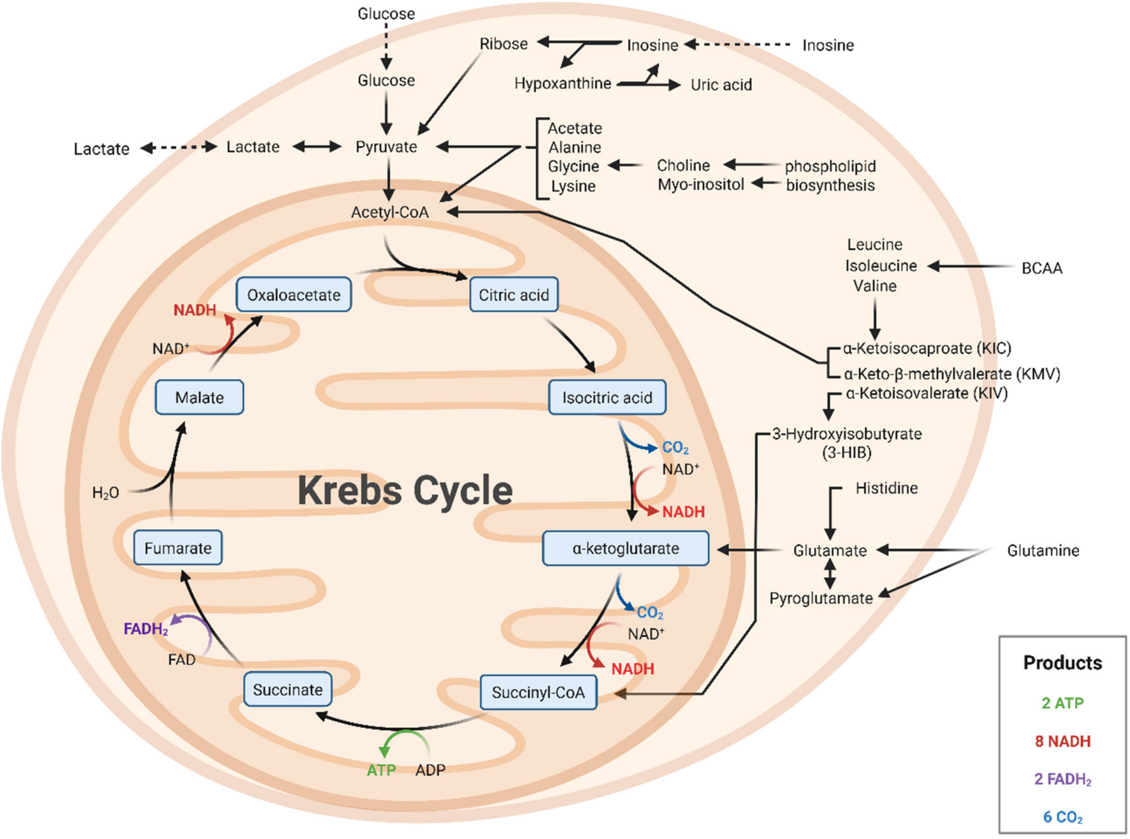

Figure S10: Schematic drawing of the TCA cycle and relevant metabolites that were regulated during serum dependency experiments and inflammation experiments [1,2,11–20,3,21,22,4–10].

References:

1. Kim, B.; Li, J.; Jang, C.; Arany, Z. Glutamine Fuels Proliferation but Not Migration of Endothelial Cells. *EMBO J.* 2017, *36*, 2321–2333, doi:10.15252/embj.201796436.
2. Leung Wai Sum, S.; Shi, Y. The Glycolytic Process in Endothelial Cells and Its Implications. *Acta Pharmacol. Sin.* 2022, *43*, 251–259, doi:10.1038/s41401-021-00647-y.
3. Sonnewald, U. Glutamate Synthesis Has to Be Matched by Its Degradation – Where Do All the Carbons Go? *J. Neurochem.* 2014, *131*, 399–406, doi:https://doi.org/10.1111/jnc.12812.
4. Teuwen, L.-A.; Geldhof, V.; Carmeliet, P. How Glucose, Glutamine and Fatty Acid Metabolism Shape Blood and Lymph Vessel Development. *Dev. Biol.* 2019, *447*, 90–102, doi:https://doi.org/10.1016/j.ydbio.2017.12.001.
5. Yetkin-Arik, B.; Vogels, I.M.C.; Nowak-Sliwinska, P.; Weiss, A.; Houtkooper, R.H.; Van Noorden, C.J.F.; Klaassen, I.; Schlingemann, R.O. The Role of Glycolysis and Mitochondrial Respiration in the Formation and Functioning of Endothelial Tip Cells during Angiogenesis. *Sci. Rep.* 2019, *9*, 12608, doi:10.1038/s41598-019-48676-2.
6. Li, X.; Sun, X.; Carmeliet, P. Hallmarks of Endothelial Cell Metabolism in Health and Disease. *Cell Metab.* 2019, *30*, 414–433, doi:https://doi.org/10.1016/j.cmet.2019.08.011.
7. Cooper, A.J.L.; Jeitner, T.M. Central Role of Glutamate Metabolism in the Maintenance of Nitrogen Homeostasis in Normal and Hyperammonemic Brain. *Biomolecules* 2016, *6*, doi:10.3390/biom6020016.
8. KRISHNASWAMY, P.R.; PAMILJANS, V.; MEISTER, A. Activated Glutamate Intermediate in the Enzymatic Synthesis of Glutamine. *J. Biol. Chem.* 1960, *235*, PC39-40.
9. Kumar, A.; Bachhawat, A.K. Pyroglutamic Acid: Throwing Light on a Lightly Studied Metabolite. *Curr. Sci.* 2012, *102*, 288–297.
10. Orlowski, M.; Meister, A. Partial Reactions Catalyzed by γ-Glutamylcysteine Synthetase and Evidence for an Activated Glutamate Intermediate. *J. Biol. Chem.* 1971, *246*, 7095–7105, doi:https://doi.org/10.1016/S0021-9258(19)45858-4.
11. Seddon, A.P.; Zhao, K.Y.; Meister, A. Activation of Glutamate by Gamma-Glutamate Kinase: Formation of Gamma-Cis-Cycloglutamyl Phosphate, an Analog of Gamma-Glutamyl Phosphate. *J. Biol. Chem.* 1989, *264*, 11326–11335.
12. Johnson, R.J.; Gomez-Pinilla, F.; Nagel, M.; Nakagawa, T.; Rodriguez-Iturbe, B.; Sanchez-Lozada, L.G.; Tolan, D.R.; Lanaspa, M.A. Cerebral Fructose Metabolism as a Potential Mechanism Driving Alzheimer's Disease . *Front. Aging Neurosci.* 2020, *12*.
13. Kim, I.S.; Jo, E.-K. Inosine: A Bioactive Metabolite with Multimodal Actions in Human Diseases. *Front. Pharmacol.* 2022, *13*.
14. Wang, T.; Gnanaprakasam, J.N.R.; Chen, X.; Kang, S.; Xu, X.; Sun, H.; Liu, L.; Rodgers, H.; Miller, E.; Cassel, T.A.; et al. Inosine Is an Alternative Carbon Source for CD8+-T-Cell Function under Glucose Restriction. *Nat. Metab.* 2020, *2*, 635–647, doi:10.1038/s42255-020-0219-4.
15. Bjune, M.S.; Lawrence-Archer, L.; Laupsa-Borge, J.; Sommersten, C.H.; McCann, A.; Glastad, R.C.; Johnston, I.G.; Kern, M.; Blüher, M.; Mellgren, G.; et al. Metabolic Role of the Hepatic Valine/3-Hydroxyisobutyrate (3-HIB) Pathway in Fatty Liver Disease. *eBioMedicine* 2023, *91*, doi:10.1016/j.ebiom.2023.104569.
16. Holecek, M.; Siman, P.; Vodenicarovova, M.; Kandar, R. Alterations in Protein and Amino Acid Metabolism in Rats Fed a Branched-Chain Amino Acid- or Leucine-Enriched Diet during Postprandial and Postabsorptive States. *Nutr. Metab. (Lond).* 2016, *13*, 12, doi:10.1186/s12986-016-0072-3.
17. Vanweert, F.; Schrauwen, P.; Phielix, E. Role of Branched-Chain Amino Acid Metabolism in the Pathogenesis of Obesity and Type 2 Diabetes-Related Metabolic Disturbances BCAA Metabolism in Type 2 Diabetes. *Nutr. Diabetes* 2022, *12*, 35, doi:10.1038/s41387-022-00213-3.
18. Eelen, G.; de Zeeuw, P.; Treps, L.; Harjes, U.; Wong, B.W.; Carmeliet, P. Endothelial Cell Metabolism. *Physiol. Rev.* 2018, *98*, 3–58, doi:10.1152/physrev.00001.2017.
19. Koziel, A.; Woyda-Ploszczyca, A.; Kicinska, A.; Jarmuszkiewicz, W. The Influence of High Glucose on the Aerobic Metabolism of Endothelial EA.Hy926 Cells. *Pflügers Arch. - Eur. J. Physiol.* 2012, *464*, 657–669, doi:10.1007/s00424-012-1156-1.
20. Sherry, E.; Lee, P.; Choi, I.-Y. In Vivo NMR Studies of the Brain with Hereditary or Acquired Metabolic Disorders. *Neurochem. Res.* 2015, *40*, doi:10.1007/s11064-015-1772-1.

21. Shi, L.; Tu, B.P. Acetyl-CoA and the Regulation of Metabolism: Mechanisms and Consequences. *Curr. Opin. Cell Biol.* 2015, *33*, 125–131, doi:10.1016/j.ceb.2015.02.003.
22. Wong, B.W.; Marsch, E.; Treps, L.; Baes, M.; Carmeliet, P. Endothelial Cell Metabolism in Health and Disease: Impact of Hypoxia. *EMBO J.* 2017, *36*, 2187–2203, doi:10.15252/embj.201696150.
